# Alpha5 nicotine acetylcholine receptor subunit promotes intrahepatic cholangiocarcinoma metastasis

**DOI:** 10.1038/s41392-024-01761-z

**Published:** 2024-03-08

**Authors:** Yan Fu, Keyu Shen, Hao Wang, Shun Wang, Xufeng Wang, Le Zhu, Yan Zheng, Tiantian Zou, Hongfei Ci, Qiongzhu Dong, Lun-Xiu Qin

**Affiliations:** 1 Hepatobiliary Surgery, Department of General Surgery, Huashan Hospital & Cancer Metastasis Institute, Fudan University, 200040 Shanghai, China; 2 Center of Interventional Radiology and Vascular Surgery, Department of Radiology, Zhongda Hospital, Medical School, Southeast University, 87 Dingjiaqiao Road, 210009 Nanjing, China; 3 Institutes of Biomedical Sciences, Fudan University, 200032 Shanghai, China; 4 Key Laboratory of Whole-period Monitoring and Precise Intervention of Digestive Cancer, Shanghai Municipal Health Commission, Minhang Hospital, Fudan University, Shanghai, China

**Keywords:** Metastasis, Gastrointestinal cancer

## Abstract

Neurotransmitter-initiated signaling pathway were reported to play an important role in regulating the malignant phenotype of tumor cells. Cancer cells could exhibit a “neural addiction” property and build up local nerve networks to achieve an enhanced neurotransmitter-initiated signaling through nerve growth factor-mediated axonogenesis. Targeting the dysregulated nervous systems might represent a novel strategy for cancer treatment. However, whether intrahepatic cholangiocarcinoma (ICC) could build its own nerve networks and the role of neurotransmitters in the progression ICC remains largely unknown. Immunofluorescence staining and Enzyme-linked immunosorbent assay suggested that ICC cells and the infiltrated nerves could generate a tumor microenvironment rich in acetylcholine that promotes ICC metastasis by inducing epithelial-mesenchymal transition (EMT). Acetylcholine promoted ICC metastasis through interacting with its receptor, alpha 5 nicotine acetylcholine receptor subunits (CHRNA5). Furthermore, acetylcholine/CHRNA5 axis activated GSK3β/β-catenin signaling pathway partially through the influx of Ca^2+^-mediated activation of Ca/calmodulin-dependent protein kinases (CAMKII). In addition, acetylcholine signaling activation also expanded nerve infiltration through increasing the expression of Brain-Derived Neurotrophic Factor (BDNF), which formed a feedforward acetylcholine-BDNF axis to promote ICC progression. KN93, a small-molecule inhibitor of CAMKII, significantly inhibited the migration and enhanced the sensitivity to gemcitabine of ICC cells. Above all, Acetylcholine/CHRNA5 axis increased the expression of β-catenin to promote the metastasis and resistance to gemcitabine of ICC via CAMKII/GSK3β signaling, and the CAMKII inhibitor KN93 may be an effective therapeutic strategy for combating ICC metastasis.

## Introduction

Intrahepatic cholangiocarcinoma (ICC) accounts for ~5–10% of primary liver malignancies, and the incidence of ICC is increasing globally.^1^ ICC is often diagnosed at an advanced stage with a limited opportunity to undergo radical resection, and their prognosis is very dismal as a result of the lack of effective therapeutic options.^2^ So, it is needed to uncover the molecular mechanisms underlying ICC progression to develop novel therapeutic strategies.

Perineural invasion (PNI), defined as cancer cells appearing in any layer of the nerve sheath or surrounding at least 33% of nerve fiber circumference, has been reported in many solid malignancies including ICC.^3^ PNI was previously regarded as a major manifestation of the relationship between cancer cells and nerves.^4^ Recent studies revealed the crosstalk between cancer cells and nerves, where cancer cells can secret nerve growth factors (NGF) to induce the outgrowth of nerves, and in turn, nerves can liberate effector molecules to regulate the malignant phenotype of tumors cells.^5^ The concept of neural addiction in cancer has been recently proposed, referring to the complex relationship between the nervous system and tumor development, evidenced by the clinical and preclinical phenomenon that both peripheral and central nervous systems play a crucial role in controlling tumor formation and metastasis.^6^ Mounting evidence has revealed that nerves play a central role in the regulation of tumorigenesis and cancer progression. For example, surgical or pharmacological denervation of the stomach significantly inhibited the tumor initiation and progression in the stomach.^7^

Neurotrophins, such as NGF, brain-derived neurotropic factor (BDNF), and glial cellline–derived neurotrophic factor (GDNF), are proteins engaged in neuronal growth and PNI.^3^ During neural development, BDNF activates the TrKB signaling pathway to promote the outgrowth of nerves.^8^ In pancreatic ductal adenocarcinoma, tumor cells secrete BDNF to induce neurite outgrowth and promote neural infiltration.^9^ In ovarian cancer, catecholamines-mediated activation of the ADRB3/cAMP/Epac/JNK signaling pathway upregulated the expression of BDNF to promote axonogenesis.^10^ A previous study revealed that the expression of BDNF in ICC is significantly higher than that in the adjacent normal tissues, and the BDNF level is positively correlated with the incidence of PNI, suggesting that BDNF might play an important role in the process of PNI in ICC.^11^

Acetylcholine, a classical neurotransmitter normally released from the nerve ending or synthesized by various kinds of non-neuronal cells, was reported to promote the stemness property and progression of carcinomas of the thyroid and stomach.^12,13^ Acetylcholine receptors can be classified into muscarinic acetylcholine receptor (M-AChR, which belongs to G-protein coupled receptor) and nicotinic acetylcholine receptor (N-AChR, which belongs to ligand-gated ion channel receptor). Acetylcholine-mediated activation of N-AChR can induce the influx of positive ions to modulate the intracellular signal transduction implicated in the regulation of cell proliferation, differentiation and apoptosis.^14^ α5-nAChR (CHRNA5), a member of the N-AChR family, can form a complex with α4β2 receptor to significantly enhance the ability of α4β2 to penetrate calcium ions, thereby increasing the intracellular calcium concentration in the presence of acetylcholine.^15^ Previous studies have shown that the abnormal expression of CHRNA5 is closely associated with nicotine addiction and the malignant phenotype of lung cancer.^16^ However, little is known about the role of CHRNA5 in ICC.

In the present study, we found that acetylcholine, derived from parasympathetic nerves or the self-synthesis of tumor cells, can induce epithelial-mesenchymal transition (EMT) to promote ICC metastasis. Mechanistically, CHRNA5-mediated influx of Ca^2+^ in the presence of acetylcholine activates the Ca/calmodulin-dependent protein kinases (CAMKII), which further phosphorylate and inactivate GSK3β to promote the stability of β-catenin. In addition, the acetylcholine/CHRNA5 axis-mediated upregulation of β-catenin increased the expression of BDNF in ICC to induce axonogenesis, and the regenerated axons can in turn activate the acetylcholine/CHRNA5 axis in ICC, forming a positive feedback loop between ICC cells and nerves.

## Results

### Acetylcholine is demonstrated to promote metastasis of ICC by inducing EMT

PNI is regarded as an aggressive behavior of tumor cells. We found that PNI is a strong predictor for the poor prognosis of ICC after analyzing the prognostic information of 152 patients from our institute (Supplementary Fig. 1a, b). We further compared the gene expression profiles between the ICCs with PNI (PNI^+^ ICC, *n* = 6) and those without PNI (PNI^-^ ICC, *n* = 26) from TCGA database, and the Gene ontology (GO) analysis revealed that the gene set related to EMT phenotype were enriched in PNI^+^-ICCs (Supplementary Fig. 1c). Interestingly, we found the significantly higher expression level of N-cadherin and lower expression level of E-cadherin in ICC cells closer to the nerve fibers (Supplementary Fig. 1d), which indicated that nerves might promote ICC metastasis by inducing EMT. We isolated the dorsal root ganglia (DRG) from suckling mice for in vitro culture (Supplementary Fig. 1e, f), which is commonly used in vitro model to investigate the interaction between nerves and cancer cells.^17^ Co-culture with DRG significantly increased the migration and invasion ability of ICC cells and induced a downregulation of E-cadherin and upregulation of N-cadherin in ICC cells (Supplementary Fig. 1g–j). These results suggested that nerves can induce the EMT phenotype of ICC cells to promote invasion and metastasis.

**Fig. 1:**
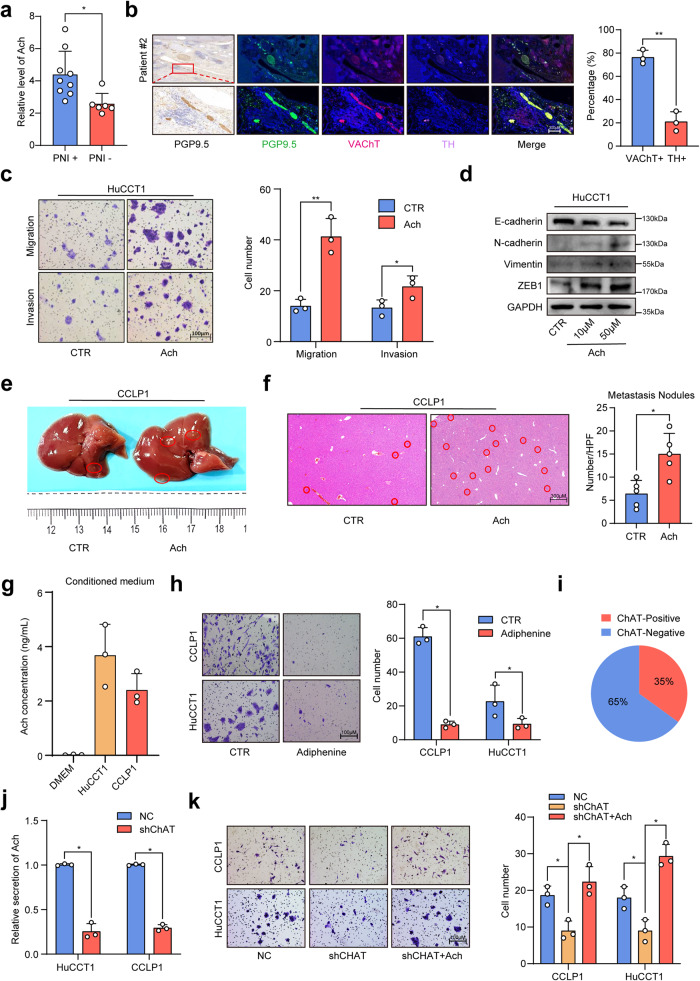
Acetylcholine-induced EMT to promote metastasis of ICC. **a** ELISA-based detection of acetylcholine in ICC tissue. **b** Immunofluorescence staining of PGP9.5, VAChT, and TH in ICC tissues. **c** Migration and invasion assays of ICC cells treated with acetylcholine. **d** WB-based detection of EMT-related genes in ICC cells treated with acetylcholine. **e** Representative images of liver metastasis of ICC cells in nude mice treated with acetylcholine. **f** H&E staining of liver tissues. **g** ELISA assay of acetylcholine in the culture supernatants of ICC cells. **h** Migration assays of ICC cells treated with adiphenine hydrochloride. **i** The results of IHC-based detection of ChAT positive rate in ICC tissues from Huashan cohort. **j** ELISA assays of acetylcholine in the culture supernatants of NC and sh-ChAT ICC cells. **k** Migration assays of NC and sh-ChAT ICC cells with or without the treatment of acetylcholine. Representative results from at least three experiments are shown. Data are shown as means ± SD. *p < 0.05, **p < 0.005

To interrogate the cellular and molecular bases by which nerves promote metastasis, we performed GO analysis to annotate the cellular functions of genes enriched in PNI^+^ ICCs from the TCGA cohort. An enrichment of “receptor-ligand interaction” was demonstrated in PNI^+^ ICCs (Supplementary Fig. 2a). Since neurotransmitter is one of the main effector ligands secreted by nerves, they might get involved in the nerve-induced EMT phenotype of ICC. Norepinephrine (NE) and acetylcholine (ACh) are the main peripheral neurotransmitters secreted by sympathetic and parasympathetic nerves, two main players in liver innervation.^18^ Detecting the content of NE and Ach in ICC by enzyme-linked immunosorbent assay (ELISA), we found that the acetylcholine level in PNI^+^ ICCs was significantly higher than that in PNI^-^ ICC, whereas no obvious difference was observed in the concentration of norepinephrine (Fig. 1a and Supplementary Fig. 2b). In addition, immunofluorescence staining assay also revealed that the majority of PGP9.5-positive nerve fibers in ICC tissues were positive for the parasympathetic marker-vesicular acetylcholine transporter (VAChT), and only a minority of nerve fibers were positive for the sympathetic marker tyrosine hydroxylase (TH) (Fig. 1b). These results hinted that PNI created a microenvironment rich in acetylcholine in ICC. In vitro assay revealed that exogenous acetylcholine significantly stimulated the EMT phenotype and enhanced the migration ability of ICC cells (Fig. 1c, d and Supplementary Fig. 2c–f), whereas no obvious effect was observed after the treatment of NE (Supplementary Fig. 2g). As reported previously,^19^ DRG could secrete acetylcholine (Supplementary Fig. 2h). Interestingly, the DRG-enhanced migration ability of ICC cells can be abrogated by nAChR inhibitor adiphenine hydrochloride, suggesting a critical role of acetylcholine signaling in the DRG-enhanced migration ability of ICC cells (Supplementary Fig. 2i, j). In vivo assay also revealed that acetylcholine treatment significantly facilitated the formation of intrahepatic metastases of CCLP1 cells (Fig. 1e, f), and adiphenine hydrochloride significantly inhibited the formation of intrahepatic metastases (Supplementary Fig. 2k, l). All these results suggested that nerves-derived acetylcholine plays a critical role in stimulating the EMT phenotype of ICC cells.

Previous studies reported that acetylcholine could also be self-synthesized by cancer cells in addition to the secretion from nerves.^20–22^ Interestingly, acetylcholine could also be detected in the culture supernatants of HuCCT1 and CCLP1 cells by ELISA (Fig. 1g), and adiphenine hydrochloride treatment significantly inhibited the in vitro migration ability of ICC cells (Fig. 1h), suggesting that ICC cells might be able to self-synthesize and secret acetylcholine to promote migration. We observed that choline acetylase (ChAT), which is critical for the synthesis of acetylcholine, was positive in ICC cell lines (Supplementary Fig. 2m), and approximately 35% (44 of 127) of ICC from our cohort were positive for ChAT (Fig. 1i). We knocked down ChAT in ICC cells (Supplementary Fig. 2n), and found that ChAT knockdown significantly decreased the secretion of acetylcholine (Fig. 1j), and inhibited the migration ability of ICC cells, which was attenuated by exogenous addition of acetylcholine (Fig. 1k).

These data suggested that acetylcholine, derived from the secretion of nerves or self-synthesis of ICC cells, induced the EMT phenotype to promote ICC metastasis.

### Blockade of acetylcholine/CHRNA5 axis inhibited the malignant phenotype of ICC in vitro and in vivo

**Fig. 2:**
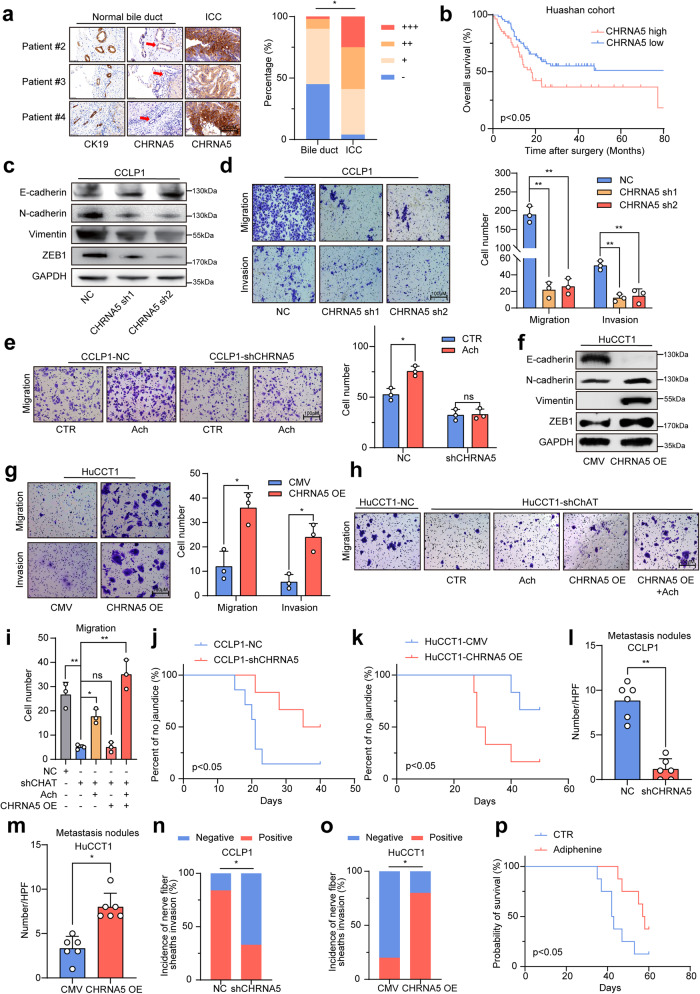
Blockade of acetylcholine/CHRNA5 suppressed ICC progression. **a** IHC staining assay of CHRNA5 in non-tumor bile duct tissues and ICC tissues. **b** The prognostic significance of CHRNA5 for ICC patients from the Huashan cohort. **c** WB of EMT-related genes in NC and sh-CHRNA5 CCLP1 cells. **d** Migration and invasion assays of NC and sh-CHRNA5 CCLP1 cells. **e** Migration assays of NC and sh-CHRNA5 CCLP1 cells treated with acetylcholine. **f** WB of EMT-related genes in CMV and CHRNA5-OE HuCCT1 cells. **g** Migration and invasion assays of CMV and CHRNA5-OE HuCCT1 cells. **h**, **i** Migration assays of NC and sh-ChAT ICC cells followed by CHRNA5 overexpression, with or without the treatment of acetylcholine. **j** Percentage of obstructive jaundice between NC and sh-CHRNA5 CCLP1 group. **k** Percentage of obstructive jaundice between HuCCT1-CMV and HuCCT1-CHRNA5 OE group. **l**, **m** Numbers of liver metastasis of ICC cells. **n**, **o** Incidence of nerve fiber sheaths invasion of ICC cells. **p** Overall survival of YAP/Akt-driven ICC mice treated with PBS or adiphenine. Representative results from at least three experiments are shown. Data are shown as means ± SD. *p < 0.05, **p < 0.005

Then, we further identified the receptor of acetylcholine involved in promoting metastasis of ICC. Firstly, we detected the mRNA expression of acetylcholine receptors in HuCCT1 and CCLP1 cell lines, and the highest level was found in CHRNA5, which was at least 64-fold higher than that of the other acetylcholine receptors (Supplementary Fig. 3a). Among these receptors of acetylcholine, only CHRNA5 expression level in tumor tissue was significantly higher than that in the adjacent non-tumor tissues from both TCGA database and GEO dataset (Supplementary Fig. 3b-d). In addition, high CHRNA5 level correlated with a poor prognosis of ICC patients from the TCGA database, but it didn’t reach statistical significance as a result of the small sample size (Supplementary Fig. 3e, f). In the patients from the author’s institute, immunohistochemical staining assays revealed a higher expression level of CHRNA5 in ICC tissues compared with the non-tumor bile duct (Fig. 2a). CHRNA5 expression level was closely associated with tumor size, lymph node metastasis, TNM stage, and serum alanine aminotransferase (ALT) level (Table 1), and ICC patients with higher CHRNA5 expression had significantly shorter OS and DFS (Fig. 2b and Supplementary Fig. 3g). These indicate that CHRNA5 is significantly associated with the malignant phenotype of ICC.

**Table 1:** The relationship between CHRNA5 expression level and the clinical characteristics in ICC

Clinical pathological characteristics		CHRNA5		*χ* value	*p* value
		Low	High		
Gender	Male	32	21	0.738	0.4693
	Female	39	35		
Age	≤55	33	23	0.371	0.334
	>55	38	33		
Total bilirubin	≤21	61	47	0.097	0.473
	>21	10	9		
Alanine transaminase	≤40	55	34	4.189	0.032
	>40	16	22		
γ-glutamyl transpeptidas	≤45	32	21	0.738	0.249
	>45	39	35		
Tumor Size	≤6	36	37	3.025	0.059
	>6	35	19		
Lymph node metastasis	Negative	49	20	13.991	<0.005
	Positive	22	36		
Differentiation	Well, moderate	43	36	0.185	0.404
	Poor	28	20		
TNM phase	I–II	46	16	16.435	<0.005
	III–IV	25	40		

Furthermore, we knocked down CHRNA5 expression in CCLP1 (Supplementary Fig. 3h, i) and found that it significantly inhibited the EMT phenotype of ICC cells (Fig. 2c, d and Supplementary Fig. 3j), and abrogated the acetylcholine-induced EMT phenotype of ICC cells (Fig. 2e and Supplementary Fig. 3k). CHRNA5 knockdown also attenuated the stimulatory effect of DRG on the migration ability of ICC cells (Supplementary Fig. 3l). We also overexpressed CHRNA5 in HuCCT1 (Supplementary Fig. 3m, n), and found that it could enhance the EMT phenotype of ICC (Fig. 2f, g and Supplementary Fig. 3o). Interestingly, ChAT knockdown could restrain the enhanced effect of CHRNA5 on the migration ability of ICC cells, which could be restored by exogenous addition of acetylcholine (Fig. 2h, i). All these findings indicate that acetylcholine/CHRNA5 axis plays a critical role in facilitating the migration ability of ICC cells.

To further assess the significance of acetylcholine/CHRNA5 axis in vivo, we established an orthotopic implantation model in the liver hilum using ICC cells. CHRNA5 knockdown significantly alleviated the tumor-caused obstructive jaundice in nude mice (Fig. 2j and Supplementary Fig. 4a, b). On the other hand, CHRNA5 overexpression significantly exacerbated the tumor-caused obstructive jaundice (Fig. 2k and Supplementary Fig. 4c, d). Consistently, CHRNA5 knockdown significantly decreased the numbers of liver metastases after implantation of ICC cells into the spleen of nude mice (Fig. 2l and Supplementary Fig. 4e, f), and CHRNA5 overexpression increased the number of liver metastases (Fig. 2m and Supplementary Fig. 4g, h). Then, we constructed a model of sciatic nerve invasion to further investigate the in vivo effect of acetylcholine/CHRNA5 axis on the invasion ability of ICC. The results revealed that CHRNA5 knockdown decreased the abilities of ICC cells to wrap the axons of sciatic nerve, as well as invading the nerve fiber sheaths (Fig. 2n and Supplementary Fig. 4i, j), which suggests that CHRAN5 knockdown deprives the invasion ability of ICC. On the other hand, CHRNA5 overexpression enhanced the in vivo invasion ability of ICC (Fig. 2o and Supplementary Fig. 4k, l). Furthermore, we collected paired samples from the primary and metastatic lesions of six ICC patients with lymph node metastasis. IHC staining revealed that the expression of CHRNA5 in the lymph node metastatic lesions was significantly higher than that in the primary lesions (Supplementary Fig. 4m). We also constructed the YAP/Akt-driven ICC mouse model by hydrodynamic injection of YAP and Akt plasmids through the tail vein of C57 mice (Supplementary Fig. 4n). YAP/Akt-driven tumors were positive for CK19 and negative for HNF4α (Supplementary Fig. 4o), consistent with the histopathological features of ICC. Adiphenine hydrochloride treatment significantly reduced the tumor burden (Supplementary Fig. 4p, q) and prolonged the survival time of YAP/Akt ICC mice (Fig. 2p). Adiphenine treatment also decreased the expression levels of Ki67, and inhibited the EMT phenotype in the YAP/Akt-driven ICCs (Supplementary Fig. 4r). Taken together, these findings support that acetylcholine/CHRNA5 axis plays a critical role in maintaining the malignant phenotype of ICC. Blocking this axis demonstrated inhibition of the malignant phenotype both in vitro and in vivo.

### β-catenin signaling was responsible for the malignant phenotype driven by acetylcholine/CHRNA5 axis

**Fig. 3:**
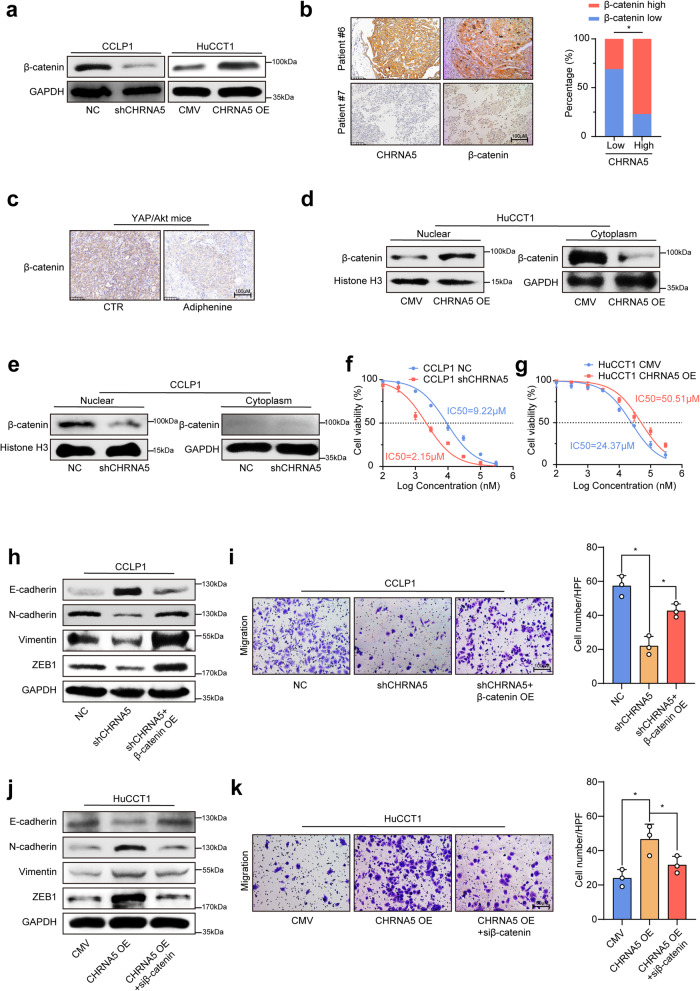
β-catenin signaling is responsible for the malignant phenotype driven by acetylcholine/CHRNA5 axis. **a** WB analysis of the expression level of β-catenin in ICC cells. **b** IHC staining of the expression level of CHRNA5 and β-catenin in clinical ICC tissue. **c** IHC assay-based detection of β-catenin in YAP/Akt ICC mice model treated with acetylcholine receptor inhibitor. **d**, **e** WB analysis of the expression level of β-catenin in the nuclear and cytoplasm of ICC cells. **f**, **g** CCK8 assay-based detection of IC50 of gemcitabine in ICC cells. **h**–**k** The effect of overexpressing or silencing β-catenin on the EMT phenotype in CHRNA5 silencing or overexpressing ICC cells. Representative results from at least three experiments are shown. Data are shown as means ± SD. *p < 0.05, **p < 0.005

Gene set enrichment analysis (GSEA)using the dysregulated genes after manipulating CHRNA5 expression in ICC cells revealed a significant enrichment of β-catenin signaling (Supplementary Fig. 5a, b). In addition, TCGA database-based GSEA also revealed that CHRNA5 was closely associated with the β-catenin activity in ICC (Supplementary Fig. 5c). Considering the critical role of β-catenin signaling in cancer metastasis, we proposed that acetylcholine/CHRNA5 axis might regulate the EMT phenotype of ICC through β-catenin. We observed that β-catenin levels in ICC cells and xenograft tissues were accordingly increased or decreased in consistent with CHRNA5 expression (Fig. 3a and Supplementary Fig. 5d). This positive correlation between CHRNA5 and β-catenin protein level was also demonstrated by IHC in clinical ICC tissues (Fig. 3b). Moreover, exogenous addition of acetylcholine could increase the expression of β-catenin (Supplementary Fig. 5e), and acetylcholine receptor inhibitor decreased the expression of β-catenin in ICC tissues from YAP/Akt mice model (Fig. 3c). Up- or down-regulation of CHRNA5 was accompanied by a corresponding increase or decrease of β-catenin protein level in the nucleus of ICC cells (Fig. 3d, e). These results suggest a regulatory role of acetylcholine/CHRNA5 axis in β-catenin expression.

Previous work reported the critical role of β-catenin in chemotherapy resistance,^23^ and we also observed that CHRNA5 knockdown significantly increased the response to gemcitabine (Fig. 3f), while CHRNA5 overexpression induced the resistance of ICC cells to gemcitabine (Fig. 3g). Furthermore, β-catenin overexpression partially neutralized the inhibitory effect of CHRNA5 knockdown on the EMT phenotype of CHRNA5-silencing CCLP1 cells (Fig. 3h, i), and the opposite effect was observed after silencing β-catenin in CHRNA5 overexpressing HuCCT1 cells (Fig. 3j, k). These results suggested that the acetylcholine/CHRNA5 axis-mediated EMT phenotype of ICC could be partially attributed to β-catenin signaling.

### Acetylcholine/CHRNA5 axis-mediated CAMKII activation suppressed GSK-3β activity to stabilize β-catenin

**Fig. 4:**
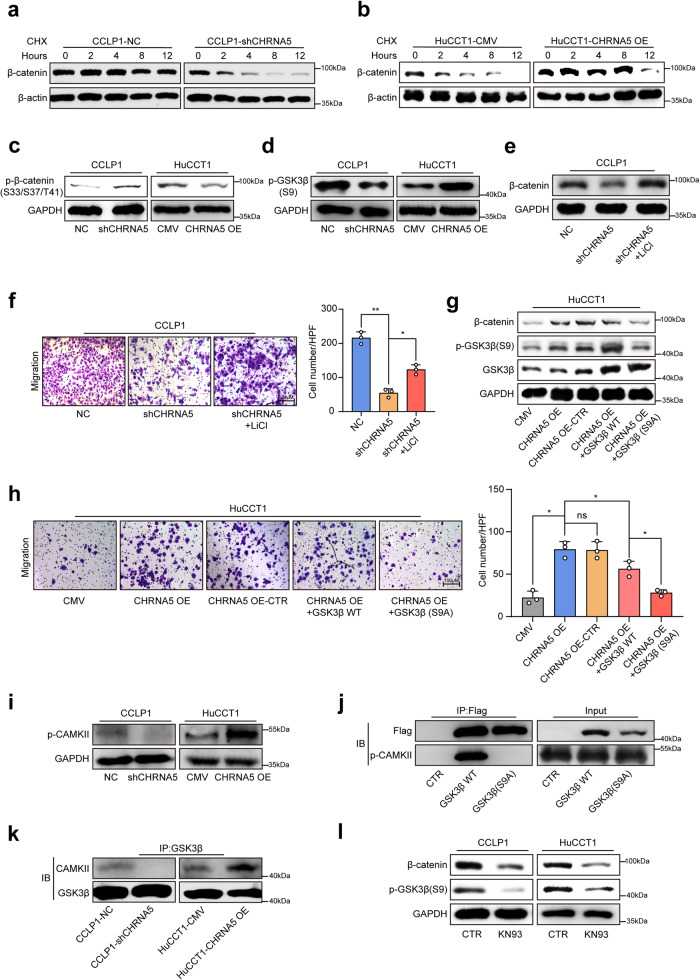
Acetylcholine/CHRNA5 axis-mediated CAMKII activation suppresses GSK-3β activity to stabilize β-catenin. **a**, **b** WB-based detection of the half-life of β-catenin in CHRNA5 silencing or overexpressing ICC cells treated with CHX. **c**, **d** WB-based detection of p-β-catenin and p-s9-GSK3β in CHRNA5 silencing or overexpressing ICC cells. **e** WB-based detection of β-catenin expression level in CHRNA5 silencing ICC cells treated with GSK3β inhibitor LiCl. **f** Transwell assay-based detection of the migration ability in CHRNA5 silencing ICC cells treated with GSK3β inhibitor LiCl. **g** WB-based detection of β-catenin expression level in CHRNA5 overexpressing ICC cells in the condition of overexpressing WT-GSK3β or mutant-GSK3β(S9A). **h** Transwell assay-based detection of the migration ability in CHRNA5 overexpressing ICC cells in the condition of overexpressing WT-GSK3β or mutant-GSK3β(S9A). **i** WB-based detection of p-CAMKII expression level in CHRNA5 silencing or overexpressing ICC cells. **j** WB-based detection of CAMKII coprecipitated with GSK3β. **k** WB-based detection of CAMKII coprecipitated with GSK3β in CHRNA5 silencing or overexpressing ICC cells. **l** WB-based detection of β-catenin and p-s9-GSK3β expression level in ICC cells treated with KN93. Representative results from at least three experiments are shown. Data are shown as means ± SD. *p < 0.05, **p < 0.005

We next explored how the acetylcholine/CHRNA5 axis regulates β-catenin expression, and observed that up- or down-regulation of CHRNA5 had no significant effect on the mRNA expression of β-catenin (Supplementary Fig. 6a, b). Then, we detected the β-catenin protein levels in ICC cells treated with cycloheximide (CHX) at different time points, and found that down-regulation of CHRNA5 markedly shortened the half-life of β-catenin (Fig. 4a), whereas CHRNA5 overexpression significantly extended the half-life of β-catenin (Fig. 4b). Furthermore, the treatment of proteasome inhibitor MG132 markedly restored the β-catenin expression in CCLP1-shCHRNA5 cells (Supplementary Fig. 6c). These indicate that CHRNA5 might regulate the degradation of β-catenin in a proteasome-mediated pathway.

Since phosphorylation of β-catenin at T41, S33, or S37 mediated by GSK3β plays an important role in promoting the ubiquitination and degradation of β-catenin,^24^ we next investigated whether CHRNA5 is involved in the regulation of β-catenin phosphorylation. CHRNA5 was demonstrated to participate in the regulation of β-catenin phosphorylation at T41, S33, S37 (Fig. 4c) and GSK3β activity (Supplementary Fig. 6d, e). Up- or down-regulation of CHRNA5 resulted in a corresponding increase or decrease of GSK3β Ser9 phosphorylation (Fig. 4d). These suggest that CHRNA5 could regulate the activity of GSK3β by inducing its phosphorylation at Ser9. We also observed that LiCl, a GSK3β inhibitor, could attenuate the inhibitory effects of CHRNA5 knockdown on β-catenin activity and migration ability of ICC (Fig. 4e, f). We further overexpressed mutant GSK3β(S9A) protein, a constitutively active form of GSK3β with a mutation of serine to alanine, in CHRNA5-overexpressing HuCCT1 cells, and observed that the GSK3β(S9A) protein exhibited a more significant inhibitory effect on the β-catenin expression and migration ability of ICC cell compared with the wild-type GSK3β protein (Fig. 4g, h). All these suggest that CHRNA5 inactivates GSK3β by inducing its phosphorylation at Ser9, which further increases the stability of the β-catenin protein and promotes ICC metastasis.

Using co-immunoprecipitation assay and mass spectrometry assays, we identified CAMKII to be a candidate protein interacting with GSK3β (Supplementary Fig. 6f). Since CAMKII has serine kinase activity, and GSK3β inactivation was caused by its Ser9 phosphorylation, we speculated that CAMKII might play a significant role in acetylcholine/CHRNA5 axis-mediated GSK3β Ser9 phosphorylation. CAMKII activity is closely associated with the intracellular Ca^2+^ concentration, and the acetylcholine/CHRNA5 axis markedly increased the intracellular Ca^2+^ concentration (Supplementary Fig. 6g). Furthermore, CHRNA5 down- or upregulation induced a significantly decreased or increased activity of CAMKII (Fig. 4i; Supplementary Fig. 6h), and adiphenine hydrochloride treatment significantly decreased p-CAMKII level in YAP/Akt ICC mice (Supplementary Fig. 6i). In addition, co-immunoprecipitation assay demonstrated that CAMKII could interact with wild-type GSK3β rather than Ser9-mutant GSK3β(S9A) (Fig. 4j). The amount of CAMKII protein binding with GSK3β was significantly decreased by knockdown of CHRNA5 and increased by CHRNA5 overexpression (Fig. 4k). Consistently, CAMKII inhibitor KN93 significantly decreased the expression of p-GSK3β(S9) and β-catenin in ICC (Fig. 4l), and markedly inhibited the EMT phenotype of ICC (Supplementary Fig. 6j, k). All these findings indicate that the acetylcholine/CHRNA5 axis could activate CAMKII, which suppressed GSK-3β activity to stabilize β-catenin.

### Acetylcholine/CHRNA5 axis promoted the secretion of BDNF in ICC to induce axonogenesis

**Fig. 5:**
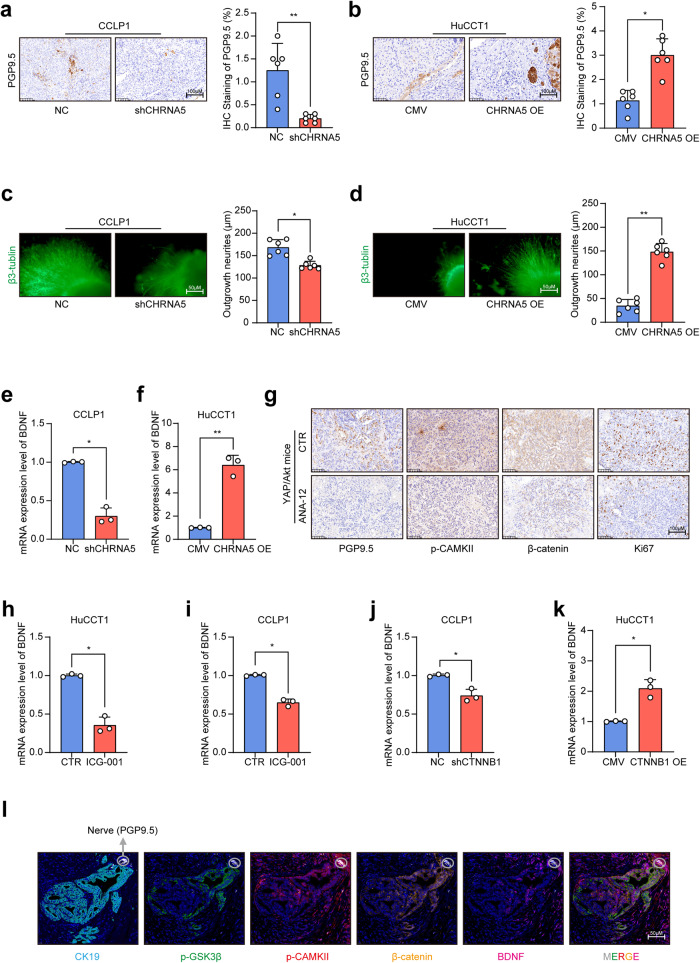
Acetylcholine/CHRNA5 axis induced axonogenesis through increasing the expression of BDNF in ICC. **a**, **b** IHC detection of PGP9.5 in CHRNA5 silencing or overexpressing ICC cells xenografts. **c**, **d** Immunofluorescence staining of β3-Tublin-based detection of nerve outgrowth of DRG cocultured with conditioned medium of CHRNA5 silencing or overexpressing ICC cells. **e**, **f** qRT-PCR-based detection of BDNF in CHRNA5 silencing or overexpressing ICC cells. **g** IHC-based detection of PGP9.5, p-CAMKII, β-catenin and Ki67 in YAP/Akt mice treated with ANA-12. **h**, **i** qRT-PCR-based detection of BDNF mRNA expression in ICC cells treated with ICG-001. **j**, **k** qRT-PCR-based detection of BDNF mRNA expression in CTNNB1 silencing or overexpressing ICC cells. **l** Multiplex immunofluorescence staining of CK19, PGP9.5, P-CAMK II, P-GSK3β, β-catenin and BDNF. Representative results from at least three experiments are shown. Data are shown as means ± SD. *p < 0.05; **p < 0.005

Acetylcholine signaling was previously reported to induce axonogenesis in gastric cancer.^25^ In order to explore whether acetylcholine signaling could induce axonogenesis in ICC, we further detected the expression level of PGP9.5 in ICC xenografts by IHC to evaluate the effect of acetylcholine signaling on axonogenesis of ICC, and found that up- or down-regulation of CHRNA5 caused a corresponding increase or decrease of axonogenesis in ICC xenograft tumors (Fig. 5a, b). In addition, acetylcholine receptor inhibitor significantly decreased axonogenesis in YAP/Akt mice (Supplementary Fig. 7a). Immunofluorescence co-staining assay revealed a positive correlation between CHRNA5 and PGP9.5 in ICC tissue samples (Supplementary Fig. 7b). To further validate the regulatory role of acetylcholine/CHRNA5 axis in axonogenesis in vitro, the conditioned culture medium of ICC cells was collected to treat DRG and the immunofluorescence staining assay was used to detect the axonal neogenesis of DRG. The result revealed that the ability to induce neurite outgrowth was significantly decreased in CCLP1 cells by CHRNA5 knockdown (Fig. 5c), but was enhanced by CHRNA5 overexpression in HuCCT1 cells (Fig. 5d), which indicates that acetylcholine/CHRNA5 axis participated in regulating the axonogenesis in ICC. Considering that partial ChAT-positive ICC could self-synthesize acetylcholine, ICC with high expression levels of both ChAT and CHRNA5 might be more prone to PNI for the increased axonogenesis. In both the TCGA database and Huashan cohorts, the incidence of PNI in ICC with high expression of ChAT and CHRNA5 was significantly higher than that in ICC with low expression of ChAT and CHRNA5 (Supplementary Fig. 7c, d).

Then, we further explored how acetylcholine/CHRNA5 axis regulates the axonogenesis in ICC. GO functional enrichment analysis was conducted using genes positively correlated with CHRNA5 in ICC, and BDNF was found to be ranked the second in the enriched gene set “Interaction Between Ligand and Receptor” (Supplementary Fig. 7e). The positive correlation between CHRNA5 and BDNF was further determined by cBioportal online analysis (Supplementary Fig. 7f). Since BDNF plays a significant role in axonogenesis through activating TrKB receptor,^26^ we detected the expression level of BDNF in ICC cells, and found that it was significantly reduced after knockdown of CHRNA5 (Fig. 5e; Supplementary Fig. 7g), and CHRNA5 overexpression significantly increased the BDNF level (Fig. 5f; Supplementary Fig. 7h). These results were consistent with that in xenograft tissues of ICC detected by ELISA (Supplementary Fig. 7i, j). In addition, acetylcholine receptor inhibitor significantly decreased the BDNF level in YAP/Akt ICC mice (Supplementary Fig. 7k). BDNF receptor inhibitor ANA-12 significantly abolished the ability of CHRNA5 to increase axonogenesis in ICC (Supplementary Fig. 7l), indicating a critical role of BDNF in the contribution of CHRNA5 to the enhanced axonogenesis in ICC. ANA-12 induced a significant decrease in tumor burden (Supplementary Fig. 7m), axonogenesis and the expression levels of p-CAMKII and β-catenin in YAP/Akt ICC mice (Fig. 5g). These suggested that the acetylcholine/CHRNA5 axis-mediated upregulation of BDNF promoted the outgrowth of nerves, and in turn, the nerves could further increase the activity of acetylcholine/CHRNA5 axis and its downstream CAMKII/GSK3β/β-catenin signaling, thereby forming a positive feedback loop to promote ICC progression.

Previous study has reported that β-catenin could initiate the transcriptional expression of BDNF in neurons and glial cells.^27^ Consistent with this notion, we found that β-catenin inhibitor ICG-001 significantly inhibited the expression level of BDNF in ICC cells (Fig. 5h, i). Therefore, we hypothesized that acetylcholine/CHRNA5 axis might regulate the expression level of BDNF through β-catenin. Based on the TCGA database, we observed that CTNNB1 shared a positive correlation with BDNF in ICC (Supplementary Fig. 7n). Knockdown of CTNNB1 decreased the expression level of BDNF (Fig. 5j), and CTNNB1 overexpression increased the expression level of BDNF in ICC (Fig. 5k). Moreover, we also observed that CAMKII inhibitor KN93 decreased BDNF expression while GSK3β inhibitor increased BDNF expression (Supplementary Fig. 7o), suggesting a regulatory role of CAMKII/GSK3β/β-catenin signaling pathway in the expression of BDNF. Above all, these results indicated that the acetylcholine/CHRNA5 axis-mediated β-catenin upregulation in ICC increased the expression of BDNF to induce the outgrowth of nerves, and in turn nerves further activated the acetylcholine/CHRNA5 axis in ICC, forming a positive feedback loop between ICC cells and nerves to promote the progression of ICC. To further consolidate our conclusion, we conducted multiple immunofluorescence analyses to explore the relationship between the expression of p-CaMKII, p-GSK3β, β-catenin, BDNF and PGP9.5 in human ICC specimens. The results revealed that the expression of p-CaMKII, p-GSK3β, β-catenin, and BDNF was significantly higher in ICC cells closer to nerves (Fig. 5l), suggesting the potential role of nerves in mediating the activation of CAMKII/GSK3β/β-catenin signaling in ICC and increasing the expression of BDNF that could further induce the outgrowth of nerves.

### CAMKII inhibitor KN93 and gemcitabine combination therapy exhibited an enhanced anti-tumor activity in ICC

**Fig. 6:**
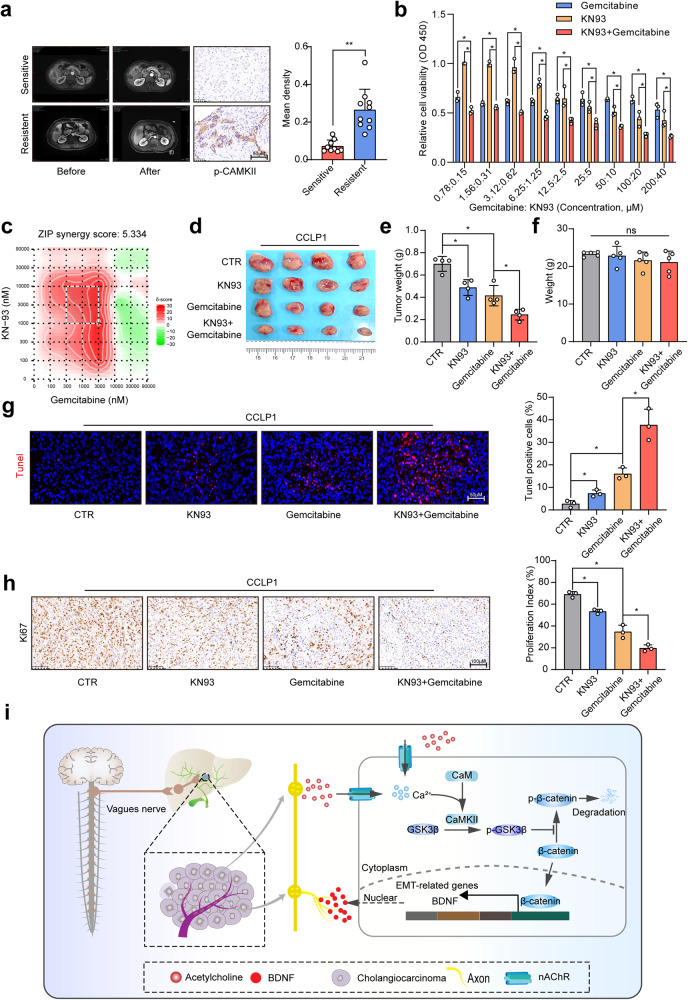
The combination of CAMKII inhibitor KN93 and gemcitabine exhibited an enhanced anti-tumor activity in ICC. **a** Representative MRI images and IHC staining of p-CAMKII in patients receiving gemcitabine-based chemotherapy. **b** CCK8 assay-based detection of inhibitory effect of KN93 and gemcitabine on CCLP1 cells. **c** Synergy score of the combination of gemcitabine and KN-93 for CCLP1. **d**, **e** In vivo detection of the therapeutic effect of KN93 and gemcitabine combined treatment. **f** The weight of nude mice from CTR, KN93, gemcitabine, KN93 and gemcitabine combined treatment group. **g** Tunel-based detection of apoptosis in CCLP1 xenograft from different groups. **h** IHC-based detection of Ki67 in CCLP1 xenograft from different groups. **i** The abstract figure of the regulatory role of acetylcholine/CHRNA5 axis in ICC: acetylcholine, derived from nerve fibers ending or cancer cells, could activate nAChR to induce the influx of Ca2+, and Ca2+-mediated CAMKII activation further phosphorylate and inactivate GSK3β, increasing the activity of β-catenin in ICC. Enhanced β-catenin activity increased the migration ability and resistance to gemcitabine in ICC. Enhanced β-catenin activity also increased the generation of BDNF to stimulate the axonogenesis, forming an Acetylcholine/CHRNA5-BDNF feedback loop to promote tumor progression in ICC. Representative results from at least three experiments are shown. Data are shown as means ± SD. *p < 0.05; **p < 0.005

Considering the essential role of CAMKII in regulating the activity of β-catenin, a critical regulator of chemotherapy sensitivity in ICC, CAMKII activity might be closely associated with the response to chemotherapy. To further explore the potential relevance between the activity of CAMKII and gemcitabine response in ICC, we collected samples from 19 ICC patients treated with gemcitabine-based chemotherapy, among whom 9 patients had a partial response (PR), and 10 had a progression disease (PD) according to the RESIST1.1. IHC staining showed that patients with resistance to gemcitabine-based chemotherapy had a higher expression level of p-CAMKII (Fig. 6a), suggesting that CAMKII might serve as a therapeutic target in ICC. In vitro assay revealed that CAMKII inhibitor KN93 treatment could increase the sensitivity to gemcitabine (Fig. 6b), and the synergistic antitumor effect was observed between KN93 and gemcitabine (Fig. 6c). Further in vivo assay also confirmed that the combined treatment of KN93 and gemcitabine was more effective than single drug therapy (Fig. 6d, e), and no significant toxicity of these drugs was observed among the mice receiving different treatment (Fig. 6f). In addition, the combination therapy could induce the apoptosis and inhibit the expression of Ki67 to a larger extent (Fig. 6g, h). Together, these findings suggest that inhibiting CAMKII could suppress the malignant phenotype and increase the sensitivity to gemcitabine in ICC, and the combination therapy of KN93 and gemcitabine might represent a novel therapeutic strategy for ICC.

## Discussion

The newly discovered contribution of nerves to tumor initiation and progression generated an emerging area of cancer neurobiology, and there existed a considerable interest in exploring the therapeutic implication of targeting the regulatory effect of nerves on cancer growth. PNI is closely related to a poor prognosis, which might be partially attributed to a result of the nerve-derived survival signals to cancer cells.^28^ Mounting evidence suggested that nerves participated in regulating the malignant phenotype of tumor cells through paracrine of neurotransmitters.^4^ In our study, we identified the regulatory role of nerves and acetylcholine in ICC metastasis and resistance to gemcitabine.

The pioneering work by Claire Magnon et al. identified the essential role of the autonomic nerve in the initiation and progression of prostate cancer.^29^ Since then, more and more studies uncovered the regulatory role of nerves in cancer. In pancreatic cancer, sympathetic nerve-derived catecholamines promote tumor development in an ADRB2-dependent manner.^9^ In gastric cancer, acetylcholine signaling promotes tumorigenesis in an ACh muscarinic receptor3-dependent manner.^25^ Cholangiocarcinoma is regarded as a kind of neurotropic cancer, but little is known about whether nerves play a regulatory role in tumor development. In our study, we found that genes overexpressed in ICC tissues with PNI were closely associated with the EMT phenotype of ICC. ICC cells with enhanced EMT phenotype may be more prone to the invasion of nerves. However, the interesting phenomenon that ICC cells, closer to nerves, exhibited a higher expression of N-cadherin and lower expression of E-cadherin, prompted us to speculate that nerves might regulate the EMT phenotype of ICC cells. Further in vitro assay revealed that co-culture with DRG increased the migration ability of ICC cells, confirming the hypothesis that nerves could promote the migration of ICC. Acetylcholine is a common neurotransmitter secreted by parasympathetic cholinergic fibers, which was previously reported to promote tumor metastasis through acetylcholine-mediated activation of stromal type 1 muscarinic receptor.^29^ Nicotine, the main addictive component of tobacco smoke, is a ligand of nicotinic acetylcholine receptors, and was reported to promote the migration of several cancers,^30–32^ also indicating the regulatory role of acetylcholine signaling in tumor migration. Here, we found an enrichment of acetylcholine in ICC tissues with PNI. Acetylcholine promoted the migration and invasion of ICC cells, and the DRG-mediated enhanced migration ability of ICC cells was reversed by acetylcholine receptor inhibitors. These indicated that nerves can promote ICC invasion and metastasis by secreting acetylcholine. Zhao et al. reported that vagotomy could suppress gastric tumorigenesis.^33^ However, it is difficult to remove parasympathetic nerves infiltrating ICC tissues, and it is a limitation of our research that we could not directly observe the effect of denervating parasympathetic nerves on the EMT phenotype of ICC in vivo.

As a member of the nicotinic acetylcholine receptor family, CHRNA5 can form a complex with α4β2 receptor and significantly increase the ability of α4β2 receptor-mediated influx of calcium ions, thereby increasing the intracellular calcium ion concentration.^15^ Here, our study revealed that the CHRNA5-mediated influx of calcium ions made a great contribution to the role of acetylcholine in promoting ICC metastasis. Silencing CHRNA5 attenuated the stimulatory effect of acetylcholine on the migration ability of ICC cells, suggesting that acetylcholine-mediated EMT phenotype of ICC relys on CHRNA5. Jiao et al. also reported that acetylcholine promotes lung adenocarcinoma metastasis through CHRNA5,^34^ proposing the hypothesis that the regulatory role of acetylcholine/CHRNA5 axis in cancer metastasis might be a common phenomenon across pan-cancer, and further studies are needed to confirm this hypothsis. Recently, increasing number of studies reported the critical role of CHRNA5 in the progression of various kinds of tumor, such as breast, colon, prostate cancer, lung cancer, and hepatocellular carcinoma,^35–37^ suggesting a crucial role of CHRNA5-based acetylcholine signaling in tumor progression.

In addition to nerves, various kinds of non-neuronal cells could also synthesize acetylcholine. Pan et al. reported that pulmonary epithelial cells could secret acetylcholine to remodel lung pre-metastastic niche of breast cancer by enhancing NETosis under the condition of chronic stress.^38^ Recent study also reported that T cells could secret acetylcholine, and T cell-derived acetylcholine was reported to play an important role in regulating immunity.^39^ In addition to normal non-neuronal cells, cancer cells could also self-synthesize and secret acetylcholine. The ability of self-synthesizing acetylcholine has also been reported in several tumors, such as prostate cancer,^40^ and gastric cancer.^41^ Interestingly, we also observed that ICC cells could self-synthesize and secret acetylcholine, which was the underlying reason for the phenomenon that EMT phenotype of cancer cells could be modulated by CHRNA5 in vitro. ChAT is a key enzyme in the process of acetylcholine synthesis. We observed the expression of ChAT in ICC cell lines, and the positive rate of ChAT in ICC tissues is about 35% in our cohort. Silencing ChAT expression could inhibit the secretion of acetylcholine and attenuate the stimulating effect of CHRNA5 on the EMT phenotype of ICC, suggesting that auto-activation of acetylcholine/CHRNA5 axis also played a crucial role in ICC metastasis. So, our study revealed that both nerves and ICC cells could secret acetylcholine to generate an tumor microenvironment rich in acetylcholine, activating CHRNA5-based acetylcholine signaling to promote ICC metastasis. It is difficult to estimate which kind source of acetylcholine, secreted by nerves or cancer cells, played a more significant role in regulating the malignant phenotype of ICC. Actually, we observed that ChAT-positive ICC exhibited a higher incidence of PNI, which means a possibly much larger production of acetylcholine in ICC tissues. This suggested that ICC cells self-activating acetylcholine signaling could further “domesticate” parasympathetic nerves to supply more acetylcholine for ICC cells.

Little is known about the mechanism underlying the regulatory role of acetylcholine /CHRNA5 axis in regulating the malignant phenotype of cancer. A recent study reported that chronic stress could activate CHRNA5-based cetylcholine signaling to promote lung adenocarcinoma progression with the underlying mechanism of DNA methyltransferase 1-mediated DNA methylation of the promoter of fragile histidine triad (FHIT).^34^ Here, our study provided an important new insight into the mechanism of how acetylcholine/CHRNA5 axis biologically affects the malignant phenotype of ICC (Summarized in Fig. 6i). Our team previously reported the contribution of β-catenin to the regulatory role of Osteopontin in ICC metastasis.^42^ Here, we found that β-catenin also plays a critical role in the acetylcholine/CHRNA5 axis-modulated migration ability of ICC cells, further confirming the regulatory role of β-catenin in ICC metastasis. β-catenin participates in regulating the stemness property and chemotherapy resistance. Here, we also found that CHRNA5 participated in regulating the stemness property and sensitivity to gemcitabine in ICC, which might be attributed to the regulatory role of CHRNA5 in the activity of β-catenin. Cingir Koker S et al. also reported that CHRNA5 contributes to chemo-resistance by regulating BAX/BCL2 ratio in breast cancer.^43^ To further identify the underlying mechanism of how acetylcholine/CHRNA5 axis regulates the expression of β-catenin, we detected the mRNA expression level of β-catenin, and observed that CHRNA5 had no effect on β-catenin mRNA expression. Further half-life analysis confirmed our hypothesis that acetylcholine/CHRNA5 axis regulated the stability of β-catenin. It is known that phosphorylation of β-catenin at T41, S33, or S37 mediated by GSK3β plays an important role in promoting the degradation of β-catenin. Here, we found that acetylcholine/CHRNA5 axis could phosphorylate and inactivate GSK3β to promote the stability of β-catenin. However, little is known about how CHRNA5 regulates the activity of GSK3β. Using COIP and Mass Spectrometry detection, we identified CAMKII as a GSK3β-interacting protein. Actually, a previous study described the interaction between CAMKII and GSK3β in neural cells.^44^ Here, we identified the involvement of CAMKII in regulating the activity of GSK3β in ICC for the first time. CAMKII is a downstream effector of the Ca^2+^ signaling pathway. When the intracellular Ca^2+^ concentration increases, the formed Ca^2+^/CaM complex can bind to and activate CAMKII via phosphorylation at Thr286 or Thr287.^45^ The crucial role of CHRNA5 in mediating Ca^2+^ fluxes was previously reported in nerve cells.^46^ Here, we found that acetylcholine/CHRNA5 axis could also regulate the intracellular Ca^2+^ concentration in ICC. Accordingly, the CAMKII activity could also be regulated by acetylcholine/CHRNA5 axis in ICC. CAMKII is a serine/threonine protein kinase. It can phosphorylate and regulate the activity of transcription factors, enzymes and kinases, thereby modulating cell proliferation, differentiation, apoptosis and other processes.^47,48^ Here, we found that CAMKII could directly bind to and phosphorylate GSK3β(Ser9), inhibiting the activity of GSK3β. Considering the essential role of CAMKII in ICC, we assessed the effect of CAMKII inhibitor KN93 on the malignant phenotype of ICC. The results revealed that KN93 could inhibit the EMT phenotype and increase the sensitivity to gemcitabine in ICC.

In addition to exploring the regulatory role of nerves in the malignant phenotype of cancer cells, how cancer cells regulate biological behavior is also an important field of cancer neuroscience. Numerous studies confirmed that cancer cells could secret nerve growth factors to promote axonogenesis and tumor innervation. Here, we also observed that ICC cells could induce axonogenesis. The activation of acetylcholine/CHRNA5 axis could increase the expression of BDNF, which could promote axonogenesis. In turn, the increased innervation further activated the acetylcholine/CHRNA5 axis. BDNF receptor inhibitor ANA-12 could inhibit axonogenesis and decrease the activity of acetylcholine/CHRNA5 axis and its downstream target CAMKII, confirming the positive feedback loop between cancer cells and nerves in ICC. Consistently, we observed an elevated incidence of PNI in ICC tissues with high expression of ChAT and CHRNA5 compared to those with low expression of ChAT and CHRNA5. So, it is possible that self-synthesizing acetylcholine might be an initial factor for abnormal innervation in ICC.

There are some limitations of our research. The first one is the lack of direct evidence confirming that denervation of parasympathetic could inhibit ICC progression in vivo. In addition, we also did not discuss the effect of acetylcholine signaling on the immune-microenvironment of ICC. As we all know, immune-microenvironment plays a crucial role in tumor progression, and acetylcholine signaling was reported to be an important regulator of immune system,^49^ so it is possible that parasympathetic nerves also regulate ICC progression through modulating immune-microenvironment. We think it is meaningful to illustrate whether inhibiting acetylcholine signaling could remodel the immune-microenvironment to inhibit the malignant phenotype of ICC, especially the sensitivity to immunotherapy in the background of ICC entering the era of immunotherapy. We will further explore this question in our next work.

To sum up, both nerves and ICC cells could secret acetylcholine, generating a tumor microenvironment rich in acetylcholine. Acetylcholine/CHRNA5 axis-mediated Ca^2+^ influx activated CAMKII, which directly phosphorylated and inactivated GSK3β, increasing the stability of β-catenin to promote the malignant phenotype of ICC. The CAMKII inhibitor KN93 could not only inhibit the migration ability of ICC cells but also enhance their sensitivity to gemcitabine, suggesting that the combination of KN93 and gemcitabine might be a novel therapeutic strategy for ICC, especially in patients with PNI.

## Materials and methods

### Clinical samples tissue

Totally, 127 paired pathologically confirmed cholangiocarcinoma and adjacent normal tissues collected from Huashan Hospital, Fudan University were used in this study. All the samples were embedded in Paraffin for immunohistochemical staining and immunofluorescence staining analysis. All of the enrolled patients signed the consent forms approved by the Institutional Review Boards of Huashan Hospital. The methods and experimental protocols were approved by the Institutional Review Boards of Huashan Hospital.

### Cell culture

Human cholangiocarcinoma cell lines HuCCT1 and CCLP1 were obtained from Shanghai Cell Bank and Chinese Academy of Science. All cell lines were cultured in Dulbecco’s modified Eagle’s medium (DMEM) (Gibco, USA) supplemented with 10% fetal bovine serum (FBS) (Gibco, USA) and 1% amphotericin B/penicillin/streptomycin (Beyotime, China) in a humidified incubator with 5% CO2 at 37 °C.

### Nude mouse xenograft model

All animal studies were approved by the Institutional Animal Care and Use Committee of Fudan University. For the liver metastasis model, HuCCT1 or CCLP1 cells (5 × 10^5^ cells per mouse) were slowly injected into the spleen of 6-week-old male BALB/c nude mice. After four weeks, the nude mice were euthanized by cervical dislocation and the livers were collected to observe the metastases. For the hepatic hilum implantation model, the ICC cells were dipped with a 0.5 cm×0.5 cm × 0.5 cm absorbable gelatin sponge and placed into the hepatic hilum of nude mice. After 4–6 weeks, the nude mice were euthanized to collect the livers for observation. For the sciatic nerve invasion model, the hilar tumor mass from the previous model was cut into pieces and implanted next to the sciatic nerve. The phenomena that nerve fiber sheaths get thinner or destructed was regarded as the manifestation of the invasion ability of ICC cells.

For the subcutaneous tumor model, 1 × 10^6^ ICC cells in 100 μL PBS were injected subcutaneously near the armpit of the nude mice. Five days later, the mice were randomly divided into Control group, KN93 single-drug group (intraperitoneal injection, 20 mg/kg, once/day), gemcitabine single-drug group (intraperitoneal injection, 50 mg/kg, once every 3 days), KN93 (intraperitoneal injection, 20 mg/kg, once/day) combined with gemcitabine (intraperitoneal injection, 50 mg/kg, once every 3 days) group. Mice were euthanized after 2–3 weeks of drug treatment, and the transplanted tumor mass was collected for analysis.

### Hydrodynamic injection to construct YAP/Akt ICC mice model

To construct the YAP/Akt ICC mice model, 30 μg pT3-YAPS127A, 20 μg pT3-AKT, and 4 μg sleeping beauty transposon-transposase plasmid was dissolved in 2 mL PBS and injected into the tail vein of C57 mice in 5 seconds. The mice were randomly divided into control and drug-treated groups one week later. One month later, FDG-PET/CT was performed and the mice were euthanized to collect the livers to be weighed and sent for immunohistochemical.

### Dorsal root ganglia isolation and culture

Suckling mice born within one day were anesthetized and prepared for the isolation of DRG. Under a stereo microscope, the mice’s back skin, muscle, and vertebral column were removed using micro-dissecting scissors. Then, DRG was collected into the ice-cooled DMEM culture medium using microsurgery forceps. Collected DRG was placed in a 24-well or 6-well plate and covered with Matrigel. After the solidification of Matrigel, ganglion culture medium was added. 48 h later, replace the culture medium with fresh culture medium with 10 μM cytarabine. After 48 h treatment of cytarabine, culture medium was replaced with fresh culture medium. After the nerve axons were extended, the culture medium was replaced with serum-free DMEM/F12 for 24 h, and the supernatant was collected as DGR-conditioned culture medium.

### Collection of conditioned media

Cells, reaching 80–90% confluence, were washed with PBS and incubated with serum-free DMEM/F12 medium (Gibco) at 37 °C for 24–48 h. The supernatant was obtained and passed through a 0.45 μm filter. Conditioned medium can be used immediately for treating DRGs or frozen at −20 °C until use.

### cDNA synthesis and quantitative real-time PCR

Total RNA was extracted from ICC cells or tumor samples using Trizol reagent (Invitrogen, USA) and reverse-transcribed into cDNA for qRT-PCR analysis following the manufacturer’s instructions for Prime-Script RT Reagent Kit (TaKaRa, Japan). TB Green Fast qPCR Mix (TaKaRa)-based quantitative real-time PCR (qRT-PCR) assays were conducted using the ABI7500 system according to the protocol. The results were expressed as the copy number of each gene relative to that of GAPDH. The sequences of the primers used in this study are listed in Supplementary Table 1.

### Western blotting

After being washed with PBS, ICC cells were lysed in modified RIPA buffer containing proteinase inhibitors and phosphatase inhibitors (Beyotime). Total proteins were measured with a BCA protein quantification kit (Beyotime) and boiled with loading buffer at 100 °C for 15 mins. Polyacrylamide gel electrophoresis and membrane transfer were conducted to separate the protein lysates. After being blocked with 5% non-fat milk in TBST, the membranes were incubated with primary antibodies at 4 °C overnight, followed by secondary antibodies for 1 h at room temperature. Enhanced chemiluminescence (ECL)-based detection of Immunocomplexes was conducted using the Gel Doc EZ Imager. GAPDH or β-actin was used as an internal reference. Antibodies information was summarized in Supplementary Table 2.

### Protein stability assay

ICC cells were treated with cycloheximide (MedChemExpress), and the cell protein samples treated with cycloheximide for 0, 2, 4, 8, and 12 h were collected. The protein expression level was detected by Western Blot, and the protein stability was analyzed.

### Isolation of nuclear and cytoplasmic proteins

The nuclear and cytoplasmic proteins were isolated with the Nuclear and Cytoplasmic Protein Extraction Kit according to the manufacturer’s protocols (Beyotime). Briefly, 20 μL cell deposition after centrifugation was dissolved with 200 μL cytoplasmic protein extraction agent A supplemented with PMSF. After vigorous vortex for 5 s and ice incubation for ~10–15 min, 10 μL cytoplasmic protein extraction agent B was added. Subsequently, the samples were incubated on ice for 1 min and centrifuged (12,000 × *g* 4 °C) for 5 min. The cytoplasmic proteins were gathered in the supernatants. Then, the deposition was mixed with 50 μL nuclear protein extraction agent supplemented with PMSF and incubated on ice for 30 min with vortexes for 15–-30 s every 2 min. Finally, centrifuge (12,000 × *g* 4 °C) for 10 min and the nuclear proteins were gathered in the supernatants.

### Plasmid construction and transfection

Lipofectamine 3000 (Invitrogen, USA) was used to conduct plasmid transfection when cells reached 70% confluence according to the instructions. PLKO plasmid loaded with lentiviral short hairpin RNA (shRNA) targeting CHRNA5 (CCGGGCTC GATTCTATTCGCTACATCTCGAGATGTAGCGAATAGAATCGAGCTTTTTG for sh1, and CCGGCCTGATGACTATGGTGGAATACTCGAGTATTCCACCATA GTCATCAGGTTTTTG for sh2) and control vectors (sh-NC) were used to silencing CHRNA5 expression. The PCDH plasmid loaded with CHRNA5 CDS sequence was used for CHRNA5 overexpression in ICC cells. Plasmid containing the gene of interest was then coated by lentivirus with Lipofectamine 3000 (Thermo Fisher) in 293 T cell line to generate lentivirus particles. The CCA cells to be infected were seeded in 60 mm dishes in advance. When the lentivirus solution was collected, it was added to the target cell culture dish, and an appropriate amount of fresh medium was added. 24 h after infection, the medium was replaced with fresh medium and cultured for 24 h, and puromycin was added for selection to obtain stable transgenic strains. Transfection efficiencies were determined at both the protein and mRNA levels.

### Co-immunoprecipitation assay

For exogenous protein interaction detection, CMV, GSK3β-Flag (WT), and GSK3β-Flag (S9A) plasmid were transfected into CCLP1 cells. Cells were lysed in the 800 μL IP lysis buffer and the whole-cell lysates were incubated with anti-FLAG Beads (MedChemExpress) overnight at 4 °C. The immunoprecipitates were collected after centrifuge (2800 rpm, 4 °C) for 1 min and washed 5 times with IP lysis buffer, followed by being subjected to WB analysis. For endogenous protein interactions detection, cell lysates were incubated with p-GSK3β(Ser9) antibody (Cell Signaling Technology) overnight at 4 °C. 100 μL Protein A Beads were added and incubated at 4 °C for 2 h to bind antigen-antibody complex. Then, immunoprecipitates were collected after centrifuge (2800 rpm 4 °C) for 1 min and washed 5 times with IP lysis buffer, followed by being subjected to WB.

### Cell Counting Kit-8 assays

Cell Counting Kit-8 (CCK-8) assays were performed to assess cell proliferation. Briefly, ICC cells were plated in 96-well plates at a density of 2000 cells per well and incubated at 37 °C overnight. Next, 200 μL gradient diluted drug solutions were added to the wells and incubated at 37 °C. After 48–72 h, CCK-8 reagent (MedChemExpress) diluted 1:10 with DMEM was added, and incubated at 37 °C for 1–2 h. The absorbance value of each well at OD450nm was measured using a microplate reader (Thermo Fisher) and IC50 curves were drawn with Graphpad.

### Immunofluorescent staining

ICC cells in logarithmic growth phase were digested and passaged for cell crawling. After the cells adhered, replace with the DRG-conditioned medium and continue to culture for 24 h. Cells were fixed with 4% paraformaldehyde at room temperature for about 15 min and then treated with 0.5% TritonX-100 solution for about 10 min. After rinsing with PBS, the crawling sections were blocked with 5% goat serum at room temperature for about 1 h. The different primary antibodies were incubated overnight at 4 °C. Sections were then rinsed in PBS and incubated with the corresponding secondary antibodies diluted at 1:100 in the dark for 1 h at room temperature. Nuclei were stained with DAPI (Sigma-Aldrich) diluted at 1:100 in the dark for 10 min at room temperature. The sections were then taken out from the 24-well plate, and anti-fluorescence quenching sealing solution was added dropwise to the surface where the cells were attached, followed by fluorescence microscope (Olympus) observation and photographing.

### Cell invasion and migration assays

ICC cells at the logarithmic growth phase were digested and transferred to the centrifuge tube. After centrifugation (1000 rpm 5 min), the supernatant was discarded, and cells were resuspended in 1 ml of DMEM medium (serum-free), followed by cell counting. 20,000–50,000 cells resuspended in 200 μL serum-free DMEM were seeded in a transwell chamber (8 μm pore size, Corning), 500 μL DMEM containing 20% FBS was added in the bottom of 24-well plates to induce cell migration. (If the purpose is to measure the cells’ invasion ability, 80 μL of 1:8 diluted Matrigel was spread in the transwell chamber 2 h in advance, and placed in the incubator at 37 °C to solidify). After incubating for 24 h (If measuring cells’ invasion ability, time for incubation will be extended to 48 h), the culture inserts were fixed with 4% paraformaldehyde for 20 min and stained in 0.1% crystal violet. Cells that stayed on the top of the membrane were gently scraped by a cotton swab. After the membrane was dry, the culture inserts were observed and photographed under a microscope.

### Histology and immunohistochemistry

Fresh tissue was immediately fixed in 4% paraformaldehyde for 24 h and embedded in paraffin blocks, which were sliced into 4μm thick sections for subsequent immunohistochemistry and immunofluorescence analysis. Hematoxylin and eosin (HE) staining were conducted to characterize tumor architecture. After being deparaffinized, sections were incubated with hydrogen peroxide for 10 min to block endogenous peroxidase. Antigen retrieval was performed with boiled Antigen Retrieval Solution (Tris-EDTA pH 9.0) for 15 min, after which 5% bovine serum albumin (BSA) was used to block sections for 20 min at room temperature. Sections were incubated with primary antibodies overnight at 4 °C and for 1 h at room temperature. After rinsing in PBS, sections were incubated with secondary antibodies at 37 °C for 30 min. Subsequently, slides were incubated with diaminobenzidine (DAB) as a chromogen. Sections were counterstained with hematoxylin and mounted for viewing.

### Tyramide Signal Amplification multi-colour immunohistochemistry

Sections were deparaffinized in xylene and rehydrated through an ethanol gradient ending with a distilled water wash. Endogenous peroxidase was blocked by incubation with hydrogen peroxide for 10 min. At each of the 5 cycles of staining, antigen retrieval was performed with boiled Antigen Retrieval Solution (Tris-EDTA pH 9.0) for 15 min, sections were rinsed in PBS and blocked for 20 min with 5% BSA at room temperature, and incubated with different primary antibodies overnight at 4 °C. Next, incubation with HRP Labeled secondary antibodies was performed at 37 °C for 30 min followed by Tyramide Signal Amplification (TSA) fluorophores (XTSA480, XTSA520, XTSA570, XTSA620, XTSA690) incubation for 20 min. Microwave treatment (MWT) was performed at each cycle of staining to remove the Ab TSA complex. At last, all sections were counterstained with DAPI for 10 min and mounted for viewing.

### Enzyme-linked immunosorbent assay

ELISA assays to measure acetylcholine and BDNF were performed according to manufacturer’s instructions. Briefly, the frozen tissue was placed in a mortar, liquid nitrogen was added to grind, and the powder was transferred to an EP tube and dissolved in DMEM. The solution was centrifuged at 3000 rpm for 5 min and the supernatant was collected for acetylcholine and BDNF measurement.

### Calcium concentration detection

Intracellular calcium concentration was detected using the intracellular calcium detection kit (F04 method, biorab) according to manufacturer’s instruction. Briefly, cells were washed with fresh HBSS 3 times and incubated with F04 working solution at 37 °C for 30 min. Then, cells were washed 3 times again, and resuspended with HBSS and incubated at 37 °C for 30 min. The fluorescence signal intensity of the samples at 488 nm was detected by a fluorescence microscope or a flow cytometer.

### GSK3β activity assay

GSK3β activity was detected by the GSK3β activity assay kit (GENMED) according to the manufacturer’s instructions. Briefly, 30 μl of buffer C, enzymatic solution D, reaction solution E, and substrate solution F were added to a 96-well plate, and incubated at room temperature for 3 min. Subsequently, 100 μg samples to be tested were added to the corresponding wells of the 96-well plate and the absorbance value at 340 nm was measured using a microplate reader (Thermo Fisher) at time points of 0 and 5 min.

### Liver metastasis model establishment

CCLP1 cells were injected into the spleen of mice to establish a liver metastasis model. One week later, the mice were randomly divided into the control group, acetylcholine-treated group (10 mg/kg, dissolved in 500 μl PBS and subcutaneously injected into the back of mice, daily), and adiphenine hydrochloride-treated group (60 mg/kg, intraperitoneal injection, daily). Three weeks later, all mice were euthanized and liver tissues were collected for HE analysis.

### FDG-PET/CT scan

Mice were fasted for 8 h before scanning. After injecting 120μCi 18F-FDG into the tail vein, anesthesia was induced and maintained with 2% isoflurane. 45 min later, Micro-PET/CT scanning was performed. After imaging, maximum normalized uptake values for 18F-FDG were calculated.

### RNA-seq analysis

The total RNA of ICC cells was extracted using Trizol reagent (Invitrogen) and further subjected to RNA-seq analysis. All reads were uniquely mapped to a gene to evaluate the gene expression level. The dysregulated genes (fold change ≥1.5, *p* < 0.05) further underwent GSEA analysis using DEGseq R package. All dysregulated genes were used for heat map analysis.

### TUNEL assay

Tissue sections were deparaffinized in xylene and rehydrated through an ethanol gradient ending with a distilled water wash. Sections were dropped with Tris-HCl (pH 7.4–7.8) for 12–30 min at room temperature and washed with PBS 3 times. TUNEL detection solution was prepared by mixing TdT enzyme and fluorescent labeling solution in a ratio of 5:45. The samples were added with 50 μL TUNEL detection solution and incubated at 37 °C for 1 h in the dark. Next, the sections were stained with DAPI (diluted 100 times) for 10 min in the dark and covered with an anti-quenching agent. Images were taken with a fluorescence microscope.

### Statistical analysis

Statistical analysis of data was performed by GraphPad Prism 8. The experiment was repeated at least three times, and the experimental results were expressed as the mean ± standard error. Pearson correlation analysis was used for correlation analysis. The Kaplan–Meier log-rank test was performed to draw survival curves and make comparisons between groups. *p* value < 0.05 was considered to be statistically significant.

### Supplementary information

Supplementary material

## Data Availability

All data generated or analyzed during this study are included in this published article and its supplementary information files. The Cancer Genome Atlas (TCGA) datasets referenced in the study are available in a public repository from the cBioPortal website (https://www.cbioportal.org/). Public RNA-seq datasets of CCA analyzed in the study are available in Gene Expression Omnibus under accession no. GSE26566 and GSE76297.
