# Supplementary material for: Alpha5 nicotine acetylcholine receptor subunit promotes intrahepatic cholangiocarcinoma metastasis
Source: Signal Transduct Target Ther. 2024 Mar 8;9:63. doi: 10.1038/s41392-024-01761-z (PMC10920868; doi:10.1038/s41392-024-01761-z)
Supplement: Supplementary file 1 — Supplementary material [file 41392_2024_1761_MOESM1_ESM.docx]

**Supplementary Materials for**

Alpha5 Nicotine Acetylcholine Receptor Subunit Promotes Intrahepatic Cholangiocarcinoma Metastasis

Authors: Yan Fu1,4*, Keyu Shen1,2*, Hao Wang1,2*, Shun Wang1,2, Xufeng Wang1,2, Le Zhu1,2, Yan Zheng1,2, Tiantian Zou1,2, Hongfei Ci1,2, Qiongzhu Dong1,3#, Lun-Xiu Qin1,2#

Correspondence to Lun-Xiu Qin(qinlx@fudan.edu.cn) & Qiongzhu Dong(qzhdong@fudan.edu.cn)

**This PDF file includes:**

Supplementary Figure 1-7 with their legends

Supplementary Table 1-2


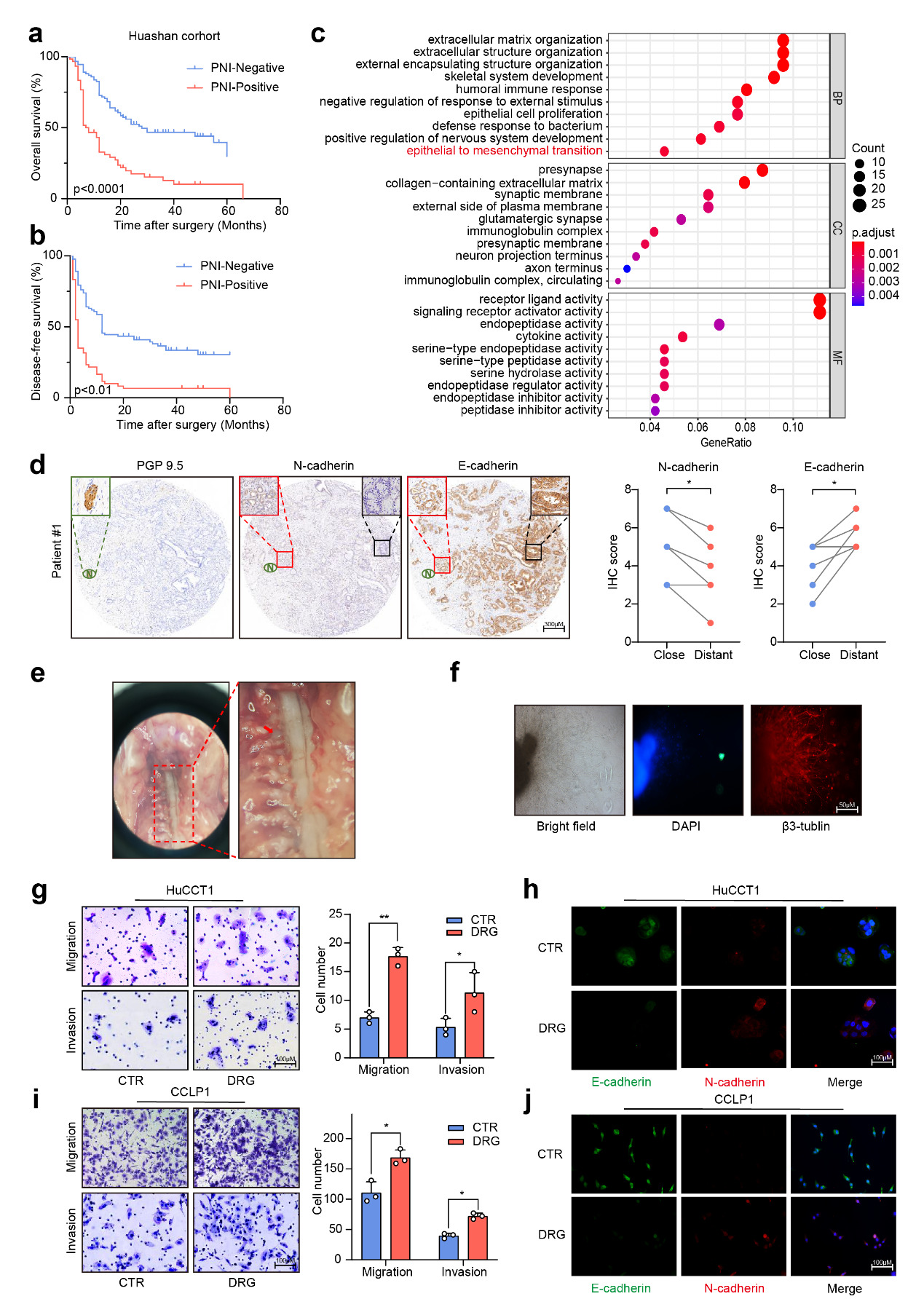


**Supplementary Figure 1. The effect of nerves on the EMT phenotype of ICC cells.**

(a, b) The prognostic significance of nerve infiltration in ICC patients. (c) GO analysis of the RNA-seq data of ICC patients from the TCGA database. (d) IHC of PGP9.5, N-cadherin, and E-cadherin in ICC tissues. The green letter N represents PGP9.5 positive nerves, red boxes represent ICC cells close to the nerves, and black boxes represent ICC cells distant from the nerves. (e) Representative images of DRG (the red arrow). (f) Immunofluorescence staining of neural filaments (β3-tubulin positive) in DRG. (g) Migration and invasion assays of HuCCT1 cells co-cultured with DRG. (h) Immunofluorescence staining of N-cadherin and E-cadherin in HuCCT1 cells co-cultured with DRG. (i) Migration and invasion assays of CCLP1 cells co-cultured with DRG. (j) Immunofluorescence staining of N-cadherin and E-cadherin in CCLP1 cells co-cultured with DRG. Representative results from at least three experiments are shown. Data are shown as means ± SD. *p<0.05, **p<0.005.


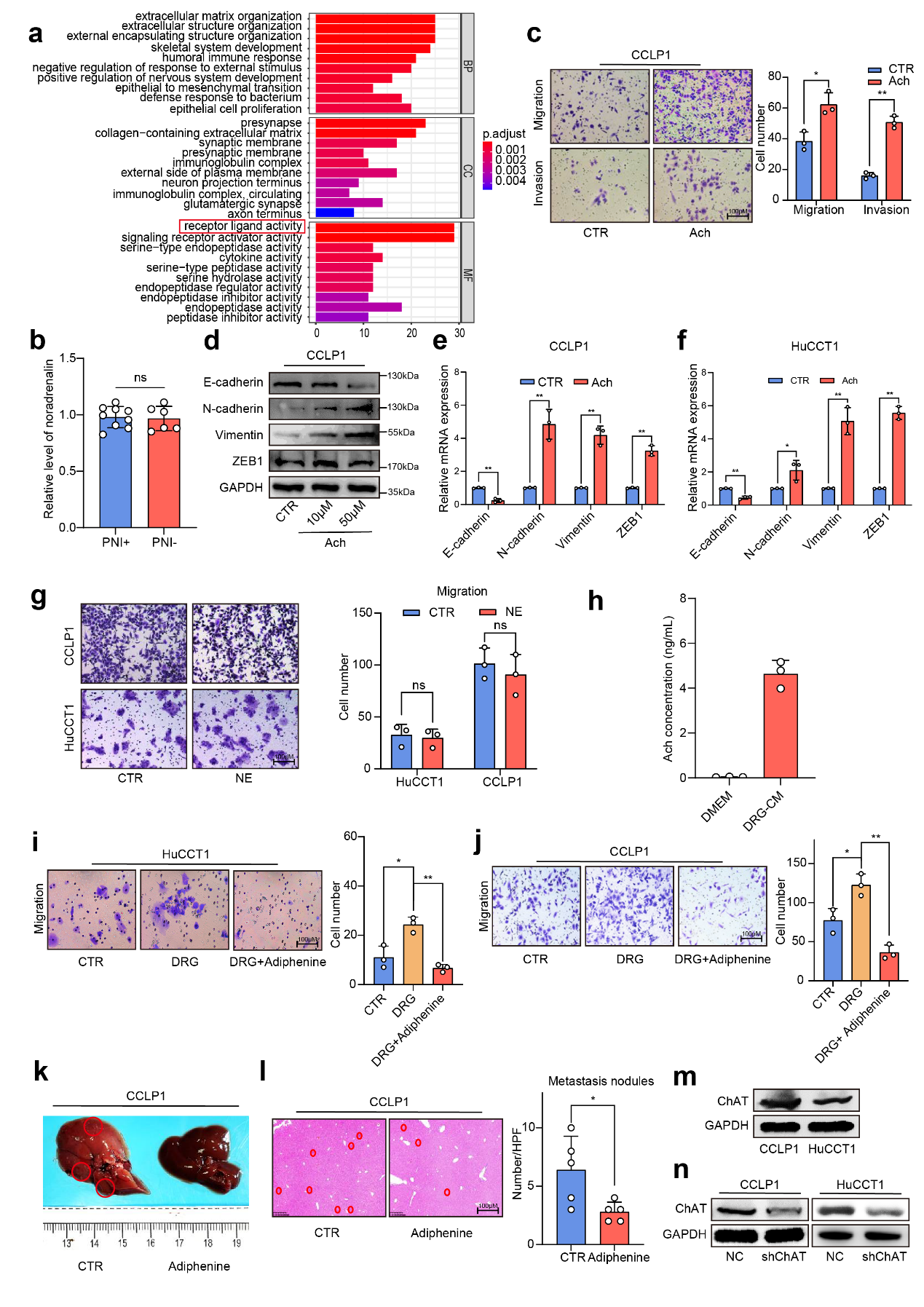


**Supplementary Figure 2. Neurotransmitter acetylcholine participated in the Nerve-driven ICC metastasis.**

(a) GO analysis of the RNA-seq data of ICC patients from the TCGA database. (b) ELISA-based detection of norepinephrine in ICC tissue. (c) Migration and invasion assays of ICC cells treated with acetylcholine. (d) WB-based detection of EMT-related genes in ICC cells treated with acetylcholine. (e, f) qRT-PCR-based detection of EMT-related genes in ICC cells treated with acetylcholine. (g) Migration and invasion assays of ICC cells treated with norepinephrine. (h) ELISA-based detection of acetylcholine in the culture supernatant of DRG. (i, j) Migration assays of ICC cells co-cultured with DRG followed by the treatment of adiphenine hydrochloride. (k) Representative images of liver metastasis of ICC cells in nude mice treated with adiphenine hydrochloride. (l) H&E staining of liver tissues. (m) WB of ChAT in ICC cell lines. (n) WB analysis of ChAT expression level in NC and sh-ChAT ICC cells. Representative results from at least three experiments are shown. Data are shown as means ± SD. *p<0.05, **p<0.005.


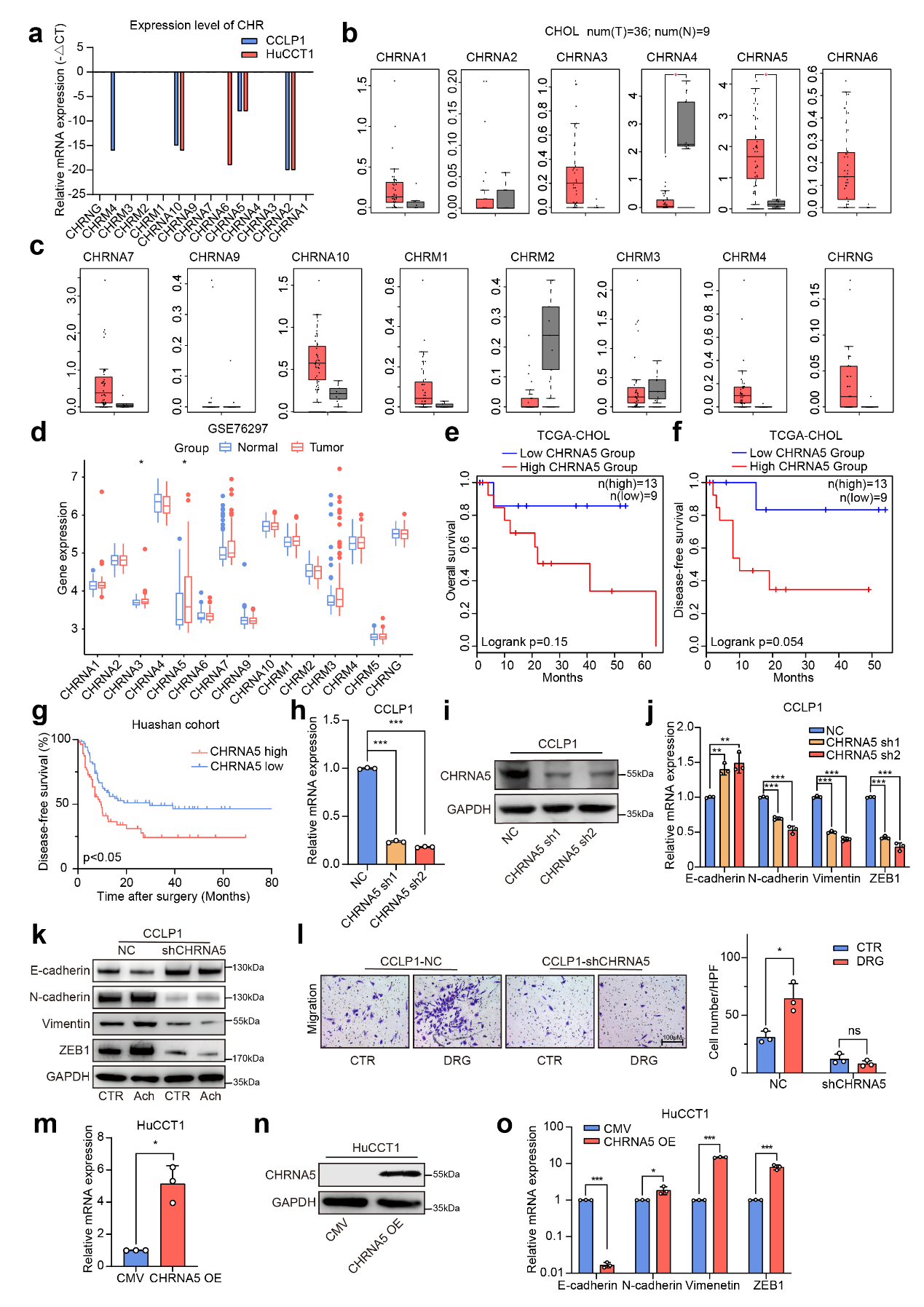


**Supplementary Figure 3. The expression and prognostic value of CHRNA5 in ICC.**

(a) qRT-PCR analysis of the mRNA expression of acetylcholine receptors in ICC cell line. (b, c) The mRNA expression of acetylcholine receptors in the ICC tissues and adjacent non-tumor tissues from the TCGA database. (d) The mRNA expression of acetylcholine receptors in the ICC tissues and adjacent non-tumor tissues from the GEO database. (e, f) The prognostic significance of CHRNA5 for ICC patients from the TCGA database. (g) The prognostic significance of CHRNA5 for ICC patients from the Huashan cohort. (h, i) qRT-PCR and WB analysis of CHRNA5 expression in NC and sh-CHRNA5 ICC cells. (j) qRT-PCR analysis of EMT-related genes in NC and sh-CHRNA5 CCLP1 cells. (k) WB analysis of EMT-related genes in NC and sh-CHRNA5 CCLP1 cells treated with acetylcholine. (l) Migration assays of NC and sh-CHRNA5 CCLP1 cells co-cultured with DRG. (m, n) qRT-PCR and WB analysis of CHRNA5 expression in CMV and CHRNA5 overexpression HuCCT1 cells. (o) qRT-PCR analysis of EMT-related genes in CMV and CHRNA5 overexpression HuCCT1 cells. Representative results from at least three experiments are shown. Data are shown as means ± SD. *p<0.05, **p<0.005.


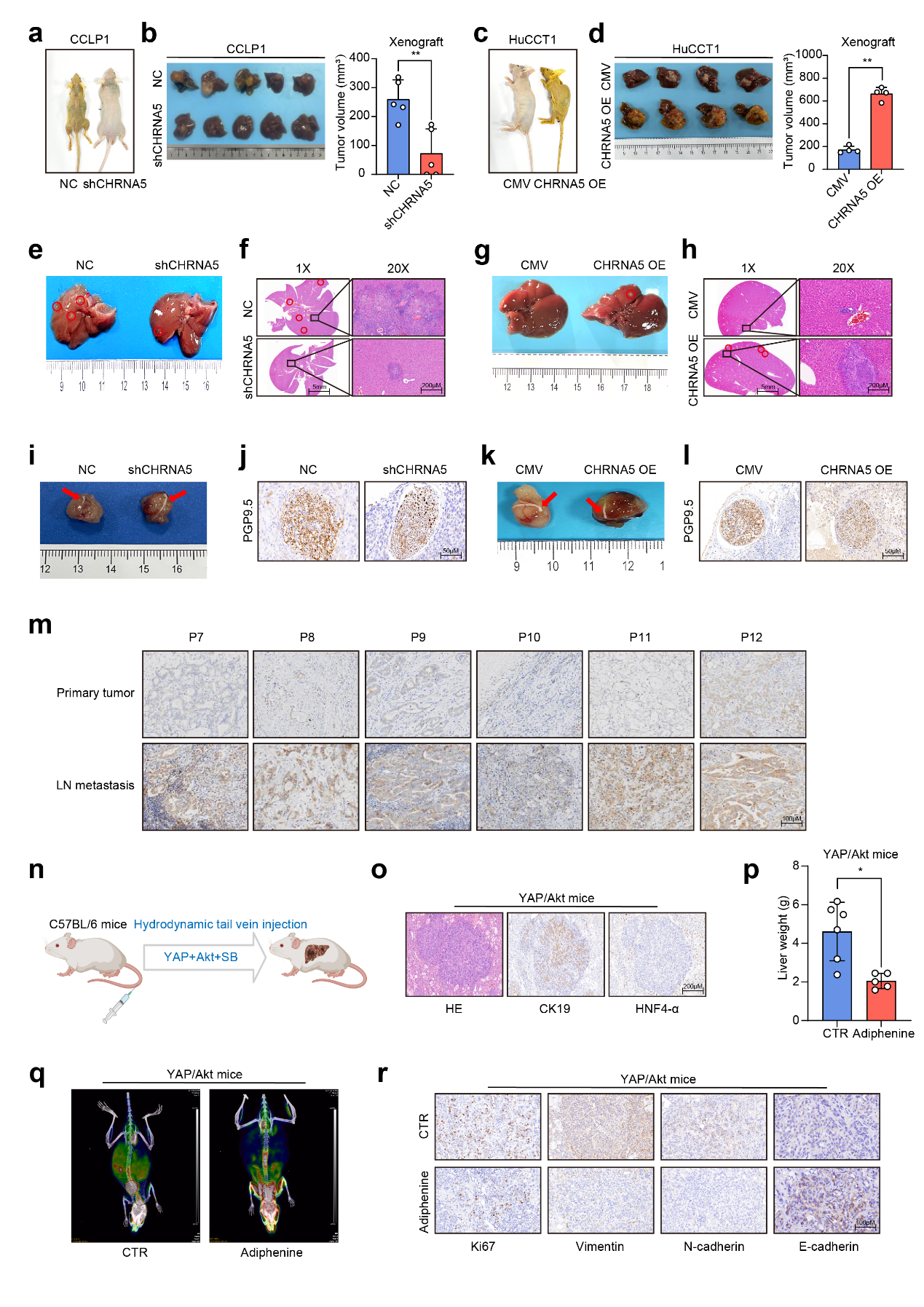


**Supplementary Figure 4. The acetylcholine/CHRNA5 axis promoted the malignant phenotype of ICC.**

(a) Obstructive jaundice after NC and sh-CHRNA5 CCLP1 cells orthotopic transplantation. (b) Orthotopic xenografts of NC and sh-CHRNA5 CCLP1 cells. (c) Obstructive jaundice after CMV and CHRNA5 OE HuCCT1 cells orthotopic transplantation. (d) Orthotopic xenografts of CMV and CHRNA5 OE HuCCT1 cells. (e-h) Representative images and H&E staining of ICC liver metastases. (i-l) Representative images and IHC staining of PGP9.5 in ectopic xenografts of ICC cells from the mouse model for perineural invasion. (m) IHC staining of CHRNA5 in primary tumor and the corresponding LN metastasis sites. (n) Diagram of the YAP/Akt-driven ICC mouse model. (o) H&E staining images and IHC of CK19 and HNF4α of the YAP/Akt-driven ICC. (p) Liver weights after the treatment with adiphenine hydrochloride in the YAP/Akt-driven ICC. (q) PET/CT scan of YAP/Akt ICC mice. (r) IHC staining of Ki67, Vimentin, N-cadherin and E-cadherin in the YAP/Akt-driven ICC. Representative results from at least three experiments are shown. Data are shown as means ± SD. *p<0.05.


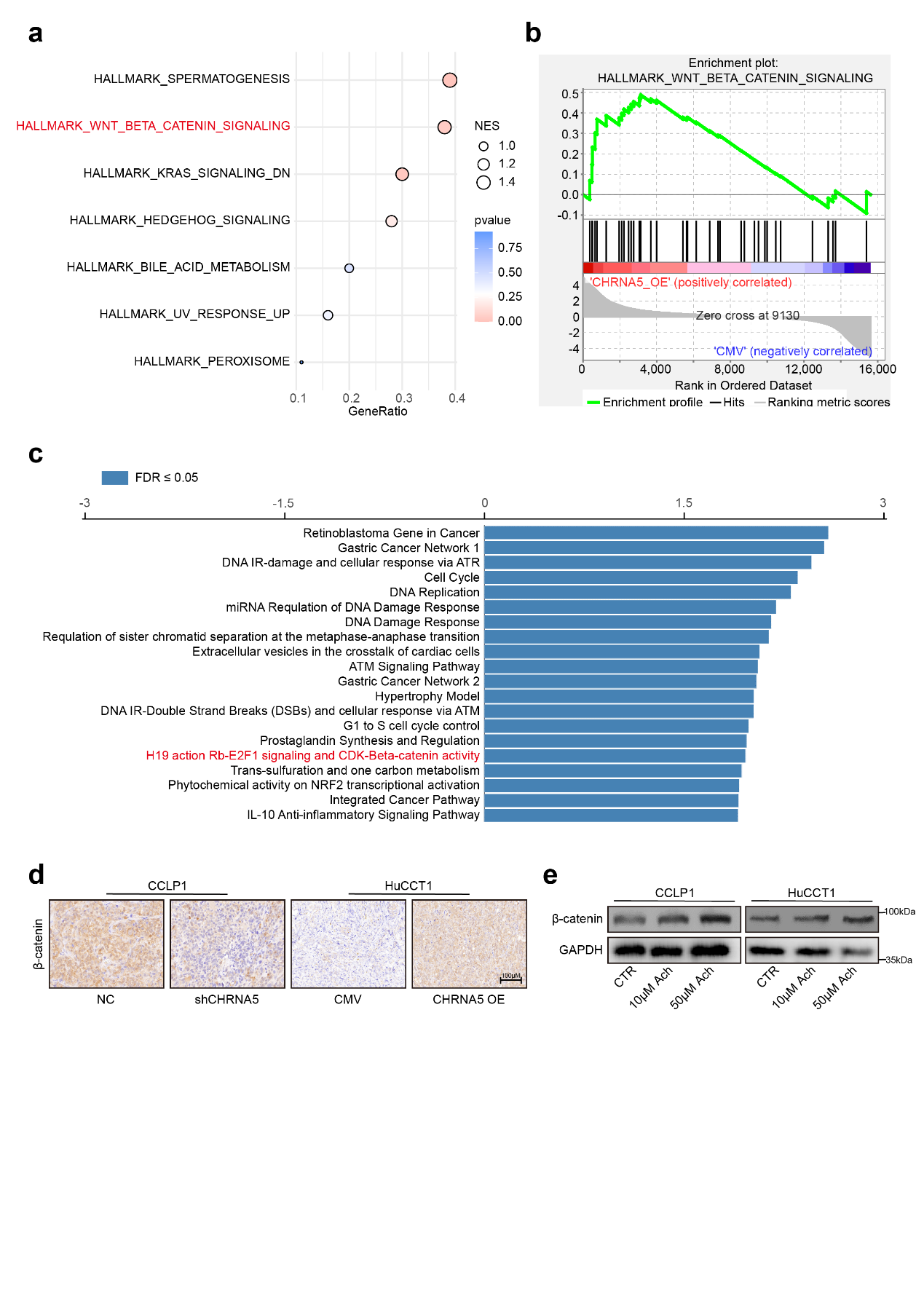


**Supplementary Figure 5. β-catenin signaling was responsible for ICC metastasis driven by acetylcholine/CHRNA5 axis**.

(a, b) GSEA analysis of β-catenin pathway with RNAseq data between HuCCT1-CMV and HuCCT1-CHRNA5 OE cells. (c) Signaling pathway enrichment analysis using genes positively correlated with CHRNA5. (d) IHC-based detection of β-catenin in CHRNA5 silencing or overexpressing ICC cell xenografts. (e) WB analysis of β-catenin in ICC cells treated with acetylcholine. Representative results from at least three experiments are shown.


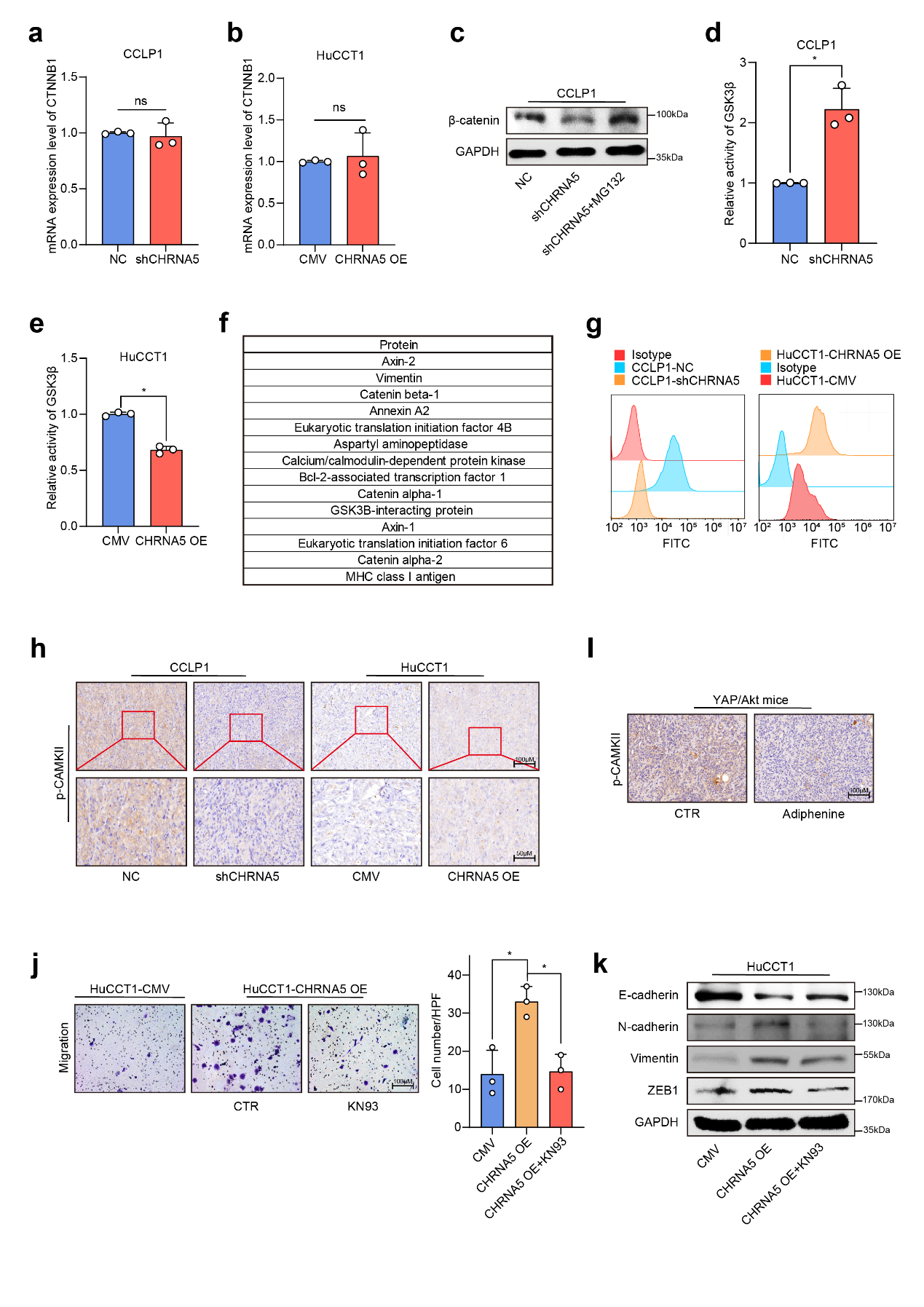


**Supplementary Figure 6. Acetylcholine/CHRNA5 axis-mediated influx of Ca2+ activates CAMKII to phosphorylate and inactivate GSK-3β.**

(a, b) qRT-PCR-based detection of mRNA levels of β-catenin in CHRNA5 silencing or overexpressing ICC cells. (c) WB-based detection of β-catenin levels in CHRNA5 silencing CCLP1 cells treated with MG132. (d, e) Detection of GSK3β activity in CHRNA5 silencing or overexpressing ICC cells. (f) Partial proteins were identified to coprecipitate with GSK3β via mass spectrometry. (g) Detection of Ca2+ concentration in CHRNA5 silencing or overexpressing ICC cells via flow cytometry. (h) IHC-based detection of p-CAMKII in CHRNA5 silencing or overexpressing ICC cells xenografts. (i) IHC-based detection of p-CAMKII in YAP/Akt ICC mice treated with adiphenine hydrochloride. (j) Migration assays of CMV and CHRNA5 OE HuCCT1 cells treated with KN93. (k) WB-based detection of EMT-associated genes in CMV and CHRNA5 OE HuCCT1 cells treated with KN93. Representative results from at least three experiments are shown. Data are shown as means ± SD. *p<0.05.


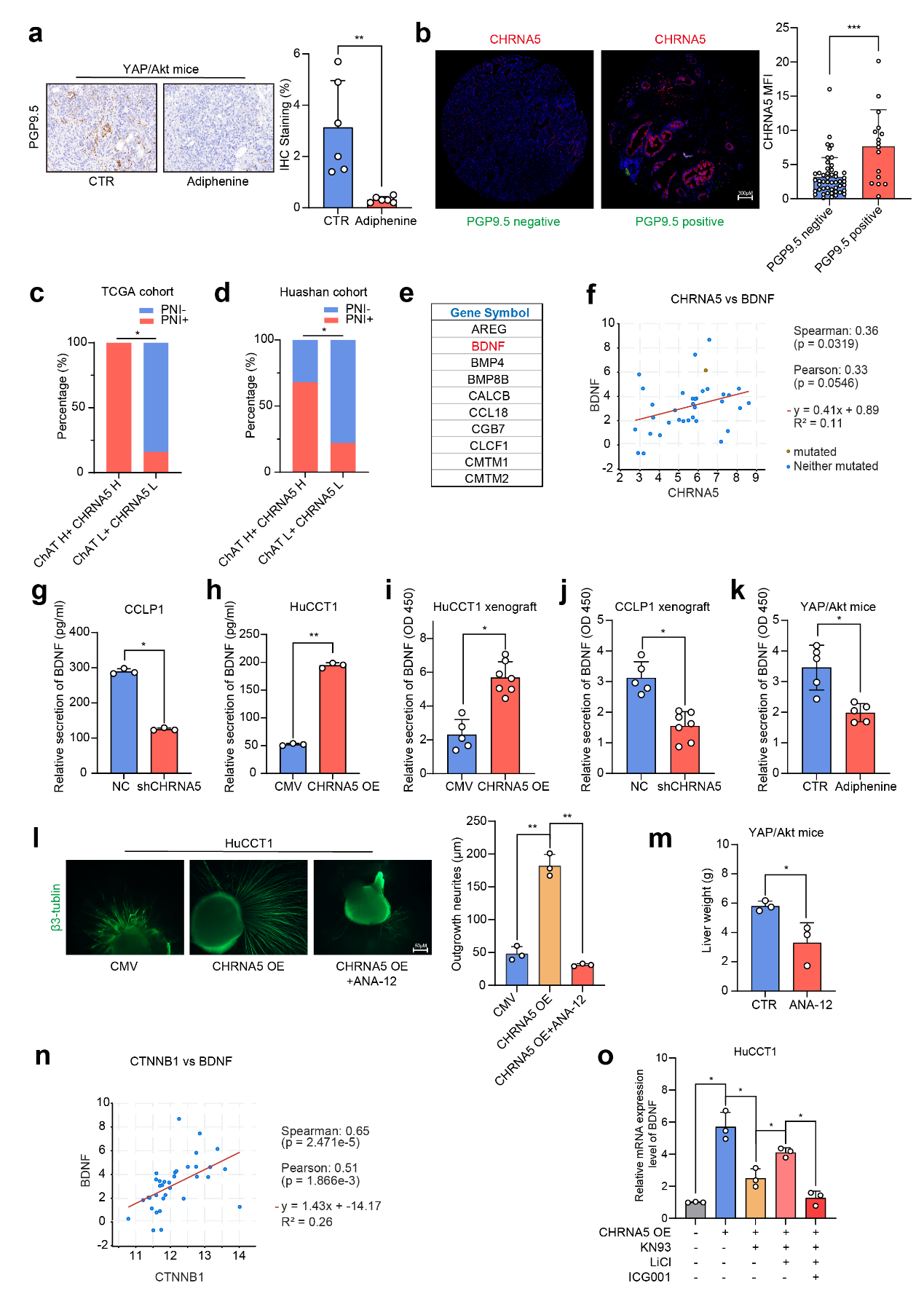


**Supplementary Figure 7.** **Acetylcholine/CHRNA5 axis increases the expression of BDNF to induce axonogenesis through regulating β-catenin activity.**

(a) IHC-based detection of the PGP9.5 expression in YAP/Akt mice treated with adiphenine hydrochloride. (b) Co-staining analysis of the relationship between CHRNA5 and PGP9.5 using ICC tissue samples. (c) Incidence of PNI in ICC from TCGA database. (d) Incidence of PNI in ICC from Huashan cohort. (e) Genes positively correlated with CHRNA5 in the enriched gene set “Interaction Between Ligand and Receptor”. (f) The correlation of CHRNA5 and BDNF in ICC from the TCGA database. (g, h) ELISA-based detection of BDNF in CHRNA5 silencing or overexpressing ICC cells. (i, j) ELISA-based detection of BDNF in CHRNA5 silencing or overexpressing ICC cells xenografts. (k) ELISA-based detection of BDNF in YAP/Akt mice treated with Adiphenine hydrochloride. (l) Immunofluorescence staining of β3-Tublin of nerves outgrowth of DRG cocultured with conditioned medium of CHRNA5 OE ICC cells treated with or without ANA-12. (m) Liver weight of YAP/Akt mice treated with ANA-12. (n) The correlation of CTNNB1 and BDNF in ICC from the TCGA database. (o) qRT-PCR-based detection of BDNF in HuCCT1 cells. Representative results from at least three experiments are shown. Data are shown as means ± SD. *p<0.05; **p<0.005.

Supplementary Table 1. Primers for qRT-PCR analysis.

| Gene | Forward | Reverse |
| --- | --- | --- |
| CHRNA1 | GCCAGAGTGCCAGTGAGAAG | CGAGGCCAGCTGAGCAA |
| CHRNA2 | GTGGAGGAGGAGGACAGA | CTTCTGCATGTGGGGTGATA |
| CHRNA3 | CAGCTGGTGAAGGTGGATGA | AGGGACACGCATGAACTCTG |
| CHRNA4 | CGTATGGGTGAAGCAGGAGT | CAGATGAGCTCGGAGGGGA |
| CHRNA5 | ACGTCTGGTTGAAACAGGAATG | TGTAGTTTGCCGGTGGAGTC |
| CHRNA6 | AAACCAATTTGTGGCTGCGT | GGAAGTCACCAACAGCATTGT |
| CHRNA7 | CCTGCACGGTAAAGCCACT | AAGCTTCCTCTGGAACTCGC |
| CHRNA9 | ACGTGGGACCGAGATCAGTA | TGAAGATTCATCATCAGCCTTGT |
| CHRNA10 | TCTCAAGCTGTTCCGTGACC | GGTTCCGTTCATCCATGTCG |
| CHRM1 | GGAGGAAGAGCAGGGTGATG | GGTGATCCCAATGAAGGCCA |
| CHRM2 | AGGACTCCTCGCTCCTTCAA | TGTCGCTAACAACCTGTCGC |
| CHRM3 | CTACCTGGAACAGGCCAACAC | GGAGATGACCCAAGCCAGAC |
| CHRM4 | TGAGGCTCCCCAGAGACAC | TGAGGCTCCCCAGAGACAC |
| CHRNG | GATGCAAAACTACGACCCCA | TCGCACCACTGCATCTCTA |
| BDNF | ACCTGAACACTTATTGCTTTG | CATTCGGCCTGAGTTTGG |
| CTNNB1 | GAGGACGGTCGGACTCCC | TCCAACTCCATCAAATCAGCTTG |
| GAPDH | TCGGAGTCAACGGATTTGGT | TGAAGGGGTCATTGATGGCA |

Supplementary Table 2. Antibodies for WB and IHC analysis.

| Antibodies | Company |
| --- | --- |
| ZEB1 | Cell Signaling Technology |
| Vimentin | Cell Signaling Technology |
| E-cadherin | Cell Signaling Technology |
| Tyrosine Hydroxylase | Cell Signaling Technology |
| N-cadherin | Cell Signaling Technology |
| p-GSK3β (Ser9) | Cell Signaling Technology |
| Anti-Flag | Cell Signaling Technology |
| β3-Tublin | Cell Signaling Technology |
| CHRNA5 | Thermo Fisher |
| SLC18A3 | Abclonal |
| p-β-catenin (S33/S37/T41) | Abclonal |
| PGP9.5 | Abcam |
| p-CAMKII (Thr 286) | Abcam |
| GAPDH | Cell Signaling Technology |
| Histone H3 | Cell Signaling Technology |
| β-catenin | Cell Signaling Technology |
| p-CAMKII (Thr 286) | GeneTex |
| Rabbit fluorescent secondary antibody (green light) | Beyotime |
| Mouse fluorescent secondary antibody (red light) | Beyotime |
| 488 goat anti-mouse fluorescent secondary antibody | Beyotime |
| Cy3-labeled goat anti-rabbit fluorescent secondary antibody | Beyotime |
| HRP-conjugate rabbit secondary antibody | Cell Signaling Technology |
| HRP-conjugate mouse secondary antibody | Cell Signaling Technology |
